# Ultrafast Electron Dynamics of a Ferrocene-Based Butadiyne-Bridged Complex

**DOI:** 10.1021/acs.jpca.5c08653

**Published:** 2026-03-16

**Authors:** Kasun C. Mendis, Jesús Valdiviezo, Susannah D. Cox, Peng Zhang, Xiao Li, Tong Ren, David N. Beratan, Igor V. Rubtsov

**Affiliations:** † Department of Chemistry, 5783 Tulane University, New Orleans, Louisiana 70118, United States; ‡ Department of Chemistry, 3065 Duke University, Durham, North Carolina 27708, United States; § Seccion Quimica, Departamento de Ciencias, 42692 Pontificia Universidad Catolica Del Peru, San Miguel, Lima 15088, Peru; ∥ Department of Physics, Duke University, Durham, North Carolina 27708, United States; ⊥ Department of Biochemistry, Duke University, Durham, North Carolina 27710, United States; # Department of Chemistry, 311308 Purdue University, West Lafayette, Indiana 47907, United States

## Abstract

Photoinduced electron transfer (ET) in alkyne-linked donor–bridge–acceptor (DBA) compounds is strongly influenced by torsional flexibility, allowing control over ET without altering the donor–acceptor distance. Here, we investigate excited-state dynamics in Fc-C4-NAP, a DBA compound featuring a ferrocene (Fc) donor, a butadiyne bridge (C4), and a 1,8-naphthalimide (NAP) acceptor. Unlike analogues DBA compounds with fully organic planar donors, Fc-C4-NAP exhibits a complex excited-state manifold. Femtosecond transient absorption (TA) measurements in the visible and mid-IR regions found three characteristic relaxation times (0.3–0.5 ps, ∼2.6 ps, and 17–20 ps) following its excitation at 402 nm, which prepares NAP-centered excited states.TD-DFT computations indicate that the acceptor-based locally excited (LE) and the charge separated (CS) diabatic states are well coupled to the Fc states associated with d-states of Fe. This bridge-mediated coupling, estimated at 200–500 cm^–1^, is strong enough to induce significant mixing of the diabatic states, which also depends strongly on the torsional angle between the NAP and the C4-bonded cyclopentadienyl ring. The spectral changes observed in the TA experiments suggest that the fast component of 0.3–0.5 ps reflects the lifetime of the bright, dominantly NAP-centered state, which relaxes predominantly to the Fc-based states. The middle component of 2.6 ps could have multiple contributions, including relaxation of the nominal CS state, vibrational cooling, and solvation. The slow decay component of ca. 20 ps corresponds to the lifetime of the lowest-energy Fc states; two Fc states of similar energies but perpendicular polarizations. The complex nature of the eigenstates, unraveled by TD-DFT analysis, results in efficient competition of the energy transfer process to the Fc-based excited states with the CS process. These results highlight the key role played by diabatic state coupling, conformational dynamics, and Fc d-orbitals in shaping the ultrafast dynamics of Fc-based DBA systems, guiding the future design of photoactive materials for solar energy and molecular electronics applications.

## Graphical abstract

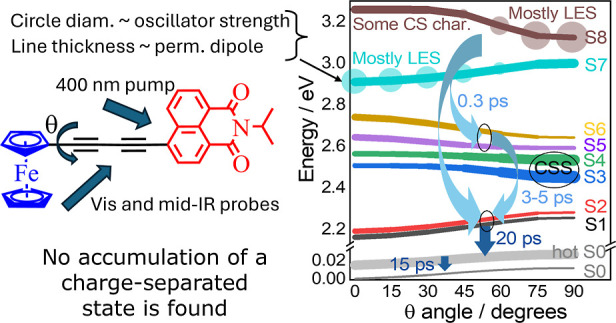

## 1 Introduction

Photoinduced electron transfer (ET) in donor-bridge-acceptor (DBA) compounds plays a central role in biological and synthetic photophysical systems. DBA compounds with conjugated bridges are particularly attractive because these bridges enhance the coupling between local electronic states, thus accelerating ET. As a result, DBAs with conjugated bridges have found wide use in applications including light-harvesting chromophores,^1–3^ optical limiting, and nonlinear absorption.^4–6^

A variety of conjugated DBA bridges have been utilized for DBA constructs, which result in D–π–A complexes,^7^ including alkenes,^8–13^ stilbenes,^14^ phenylenes,^15–17^ alkynes,^18–24^ and motifs with more complex π structures.^25–30^ Alkyne bridges feature linearity, rigidity, and compactness. These structures also allow torsional motion between the donor (D) and acceptor (A)­planes (the angle θ). Since the energy barrier for torsional motion is often small, a wide range of θ-conformers is typically accessed.^31^ The torsion angle can strongly influence bridge-mediated couplings between D and A,^32–34^ thus providing an opportunity to influence the rates of charge separation (CS) and charge recombination (CR) as well as the outcome of optical excitation.^35–38^

In some θ-conformers, for example, strong coupling between the CS state and a D- or A-localized state may strongly reduce the CS character of the lowest-energy state.^39^ Elimination of strong coupling can be achieved by increasing the energy gap between the diabatic CS and local-excited (LE) states by using a more strongly reducing electron donor, a more strongly oxidizing electron acceptor, or a more polar solvent, thus lowering the diabatic CS state energy and producing the CS state with nearly complete charge separation.^40^ Here, we studied the influence of a strong electron donor, ferrocene (Fc), on the excited state dynamics in the Fc-C4-NAP compound, where C4 is a butadiyne bridge, and NAP is *N*-isopropyl-1,8-napthalimide acceptor (Figure 1). We compare our findings with the results of our recent studies of DMA-C4-NAP with a dimethyl aniline (DMA) donor. The DMA donor studies showed a fast CS (0.63 ps) in the strongly coupled D–A conformers (θ∼15–45°) and a slow ET (4.3 ps) in the weakly coupled D–A conformers (θ∼0–15°, and 63–90°).^41^

**Figure 1:**
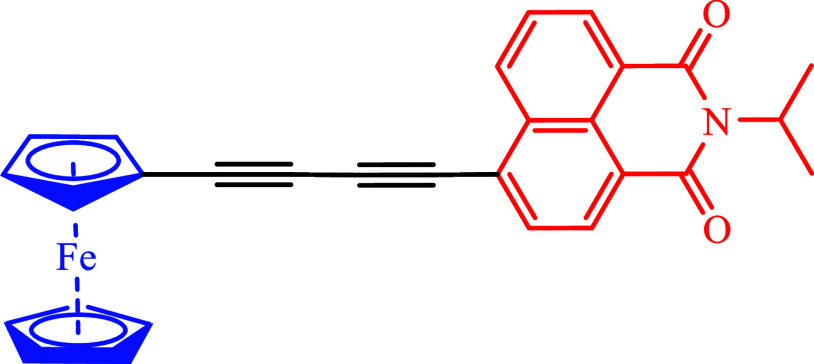
Structure of Fc-C4-NAP.

Electrochemical studies of Yuan and co-workers found that the oxidation potential for Fc/Fc^+^ in DCM of 0.46 V (measured with respect to Ag/AgCl) varies only slightly when an alkyne bridge is attached to Fc, reaching a potential of 0.5–0.6 V.^42^ Similarly, the oxidation potential of DMA (0.88–0.96 V) also increases slightly when it is attached to an alkyne moiety, reaching 1.0–1.1 V.^43^ The lower oxidation potential of Fc indicates that it is a stronger donor than DMA. In addition to being a stronger donor, the sandwich structure of Fc^44^ establishes a lower energy barrier for tortional θ motion.^45,46^ Furthermore, ferrocene-based DBA complexes are appealing because they may be suitable for future applications associated with their excellent electrochemical performance^47^ in redox probes,^48,49^ surface electrochemical applications,^50,51^ molecular electronics,^52,53^ stimuli-responsive molecular systems,^54,55^ and solar cells.^56,57^

We combine steady-state UV–vis, FTIR, and ultrafast UV/vis and UV/mid-IR transient absorption (TA) spectroscopies with TD-DFT analysis, to investigate the photoinduced dynamics of Fc-C4-NAP (Figure 1) in DCM. Despite the simplicity of the compound’s UV–vis spectrum, our findings reveal complex excited-state dynamics involving both charge separation and energy transfer pathways. Notably, the coupling between the low-lying Fc states, primarily associated with the d orbitals of Fe, and the LE and CS states produces complex state mixing. The low energy barrier for torsional motion allows access to a broad range of torsional angles and coupling of torsional motion to electron and energy transfer processes, enriching the light-driven dynamics.

### 1.1 Synthesis

The Cadiot–Chodkiewicz coupling reaction^58^ between 4-bromo-ethynyl-*N*-isopropyl-1,8-naphthalimide (BrC_2_NAP^iPr^) and ferrocene substituted ethyne (Scheme 1) was used to synthesize FcC4NAP. UV–vis, FTIR and ^1^H NMR spectroscopic techniques were used for the characterization. More details of synthesis and characterization can be found in the Supporting Information (Section S1).

**Scheme 1:**
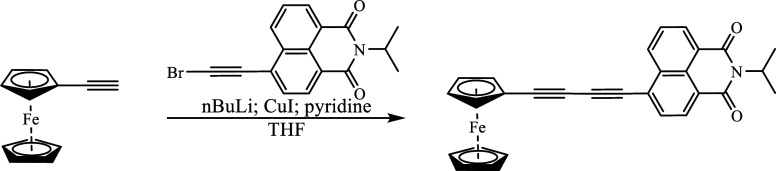
Synthesis of Fc-C4-NAP^iPr^, Denoted as Fc-C4-NAP

### 1.2 Time-Resolved Measurements

An in-house built apparatus was used for the transient UV–vis pump and Vis or m-IR probe measurements, as described recently.^41,59^ The fundamental beam at 804 nm is generated by a Ti:Sapphire fs oscillator (Vitesse, Coherent Inc.) and regenerative amplifier (Spitfire, Spectra Physics), with a 44 fs duration at a 1 kHz repetition rate. UV pulses at 402 nm of 1–1.5 μJ were generated via a frequency doubling process in a 1 mm BBO crystal; this light source provided electronic excitation of the compound. The mid-IR pulses, tunable from 1000 to 4000 cm^–1^, were generated by an optical parametric amplifier and difference-frequency generation unit featuring pulse energies of ca.1 μJ and spectral width of ca. 200 cm^–1^. The white light continuum, serving as a probe beam in the visible and near-IR regions, was generated by a portion of the fundamental beam (ca.7 μJ per pulse) in a c-cut sapphire wafer. Transient spectra in the visible and near-IR regions were measured with a CCD camera (PIXIS-100, Princeton Instrument) mounted to a monochromator (TRIAX-190, Horiba), while the mid-IR spectra were measured with a single-channel MCT detector (Infrared Associates). The sample was placed in a flow cell with 100 μm optical path length and 2 mm-thick CaF_2_ windows. The measurements were performed at room temperature (22 ± 1 °C).

### 1.3 Computational Details

DFT and TD-DFT calculations for Fc-C4-NAP were performed at the B3LYP/Def2-SVP level of theory as implemented in Gaussian 16 (Rev C.01). The polarizable continuum model (PCM) was employed to simulate the solvent effects of dichloromethane. Computations at several fixed torsion angles of 0°, 15°, 30°, 60°, 75°, and 90° between the donor (Fc) and the acceptor (NAP) were performed to track θ dependences of the spectroscopic parameters and couplings. The CAM-B3LYP, wB97X-D, and MN15 functionals were tested as well, finding B3LYP optimal for reproducing spectroscopic signals. B3LYP has been successfully used to reproduce absorption spectra of ferrocene compounds.^60–62^

## 2 Results and Discussion

### 2.1 Linear Spectroscopy Results

#### 2.1.1 UV–Vis Absorption and Emission Spectra

The UV–vis absorption spectrum of Fc-C4-NAP in DCM (Figure 2A) has two lowest energy peaks that are similar to those in the absorption spectrum of DMA-C4-NAP (Figure 2B). The peak at 385 nm is the characteristic lowest energy π–π absorption of the NAP moiety; it appears at 385 nm in DMA-C4-NAP and Si–C4–NAP and at 395 nm in Ph–C4-NAP (Figure 2B).^41^ This state is referred to as an NAP-based local excited (LE) state. Higher energy peaks at 250 and 282 nm are also assigned to the NAP-based states.

**Figure 2:**
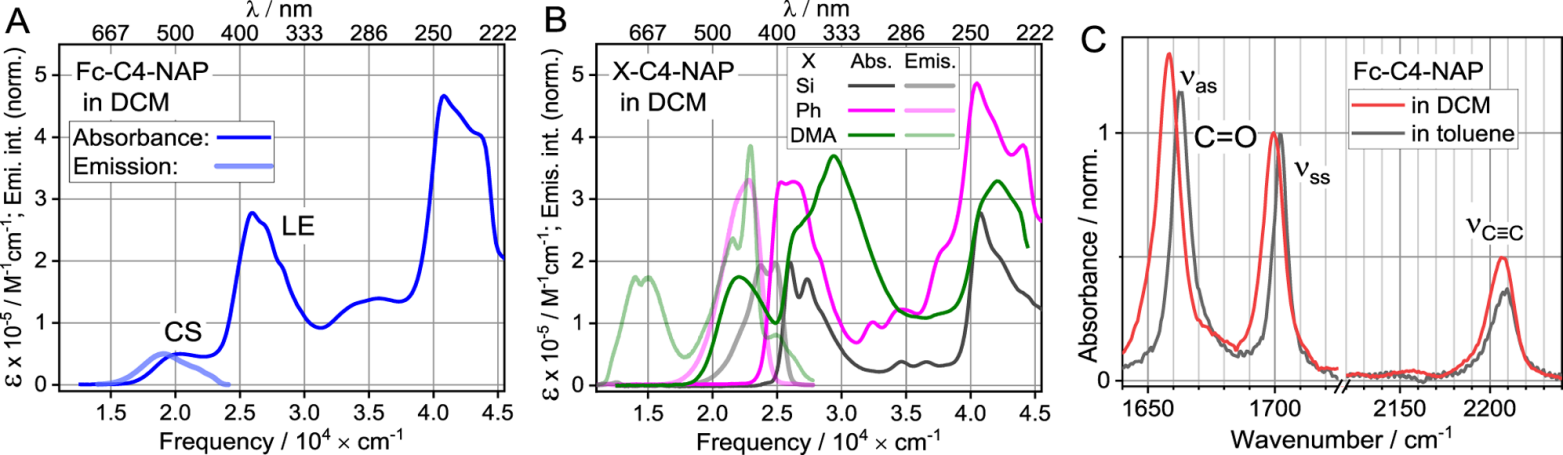
Linear absorption and emission spectra of (A) Fc-C4-NAP in DCM and (B) Si–C4–NAP, Ph–C4-NAP, and DMA-C4-NAP (λ_exc_ = 340 nm). (C). Solvent-subtracted FTIR absorption spectra of Fc0C4-NAP in DCM and in toluene.

The broad peak at ca. 500 nm (20,000 cm^–1^) is assigned to the charge-separated (CS) state (Figure 2A). Such peak was not observed in the spectra of the individual components, Fc and NAP, and in the spectra of Ph–C4-NAP and Si–C4–NAP but was observed in DMA-C4-NAP at 455 nm (22,000 cm^–1^), Figure 2B. Notice that the CS peak in Fc-C4-NAP is ca. 2000 cm^–1^ lower than that in DMA-C4-NAP, as expected due to stronger donor properties of Fc compared to DMA.^42,43^

The emission spectrum of Fc-C4-NAP (Figure 2A, light blue line) matches closely the CS state spectrum, featuring a small Stokes shift of ca. 1000 cm^–1^. Such a small Stokes shift for a highly polarized CS state in a sufficiently polar solvent (DCM) is unusual and suggests that the CS state is short-lived, not offering much time for the state solvation and internal reorganization processes. Note that the Stokes shift for the CS state for DMA-C4-NAP in DCM is ca. 8000 cm^–1^ (Figure 2B).^41^

#### 2.1.2 FTIR Spectra

Figure 2C shows selected regions of the FTIR spectrum of Fc-C4-NAP in DCM and in toluene. The symmetric C≡C stretching mode at ca. 2207 cm^–1^, denoted as ν_C≡C_, is much stronger than the antisymmetric C≡C stretching mode locates at ca. 2155 cm^–1^. The carbonyl region of the spectrum shows symmetric (∼1700 cm^–1^, ν_ss_) and antisymmetric (∼1660 cm^–1^, ν_as_) carbonyl stretching modes of NAP.^41,63^ There is a considerable solvent-dependent shift for the C=O peaks (∼5 cm^–1^ for ν_as_), which indicates significant polarization of the carbonyl bonds in the ground electronic state (GS). The solvent-dependent peak shift of ν_C≡C_ is also considerable at ca. 2 cm^–1^ (Figure 2C), indicating a polarization of the whole molecule in the GS.

### 2.2 Transient Absorption Measurements

#### 2.2.1 UV/Vis TA Results

Transient UV/vis absorption spectra at selected probe delay times, *t*, following the 402 nm excitation, are shown in Figure 3A. The 402 nm pulses excite predominantly the NAP-based LE state. The transient spectrum at short delay time (*t* = 0.2 ps) has the strongest amplitude across the visible region, peaking at ca. 475 nm. The spectrum decays rapidly at all wavelengths, except for the region of 455–520 nm, where the decay is significantly slower. We performed a decay-associated spectral analysis by fitting the data globally with a multiexponential function (Figure 3 caption). Three exponential components were required to fit the data, resulting in three characteristic times of τ_1_ = 0.5 ± 0.1 ps, τ_2_ = 2.6 ± 0.1 ps, and τ_3_ = 17 ± 0.5 ps. The amplitudes associated with each of the three components, DAS_1_(λ), DAS_2_(λ), and DAS_3_(λ), are shown in Figure 3B. Notice that the slowest component of ca. 17 ps results in a complete GS recovery within ca. 50 ps, likely indicating the presence of rapid energy transfer (EnT) processes, which were reported previously for Fc-containing compounds.^64^ The 17 ps decay time matches a typical vibrational cooling time of medium-size organic molecules to a non-hydrogen bonding solvent,^65–68^ but can also be caused by a relaxation of an electronically excited state to the GS. In other words, the state that decays with the 17 ps characteristic time could be an electronically excited state or a vibrationally hot GS. Transient UV/mid-IR spectroscopy unequivocally shows that this state is an electronically excited state (vide infra). The fastest decay component of 0.5 ps corresponds to a decay across the whole spectral region (DAS_1_ in Figure 3B). The DAS_2_, associated with the characteristic time of 2.6 ps, shows a differential shape that could be due to solvation and/or vibronic relaxation processes. It can also be due to the formation of a new state, including the CS state.

**Figure 3:**
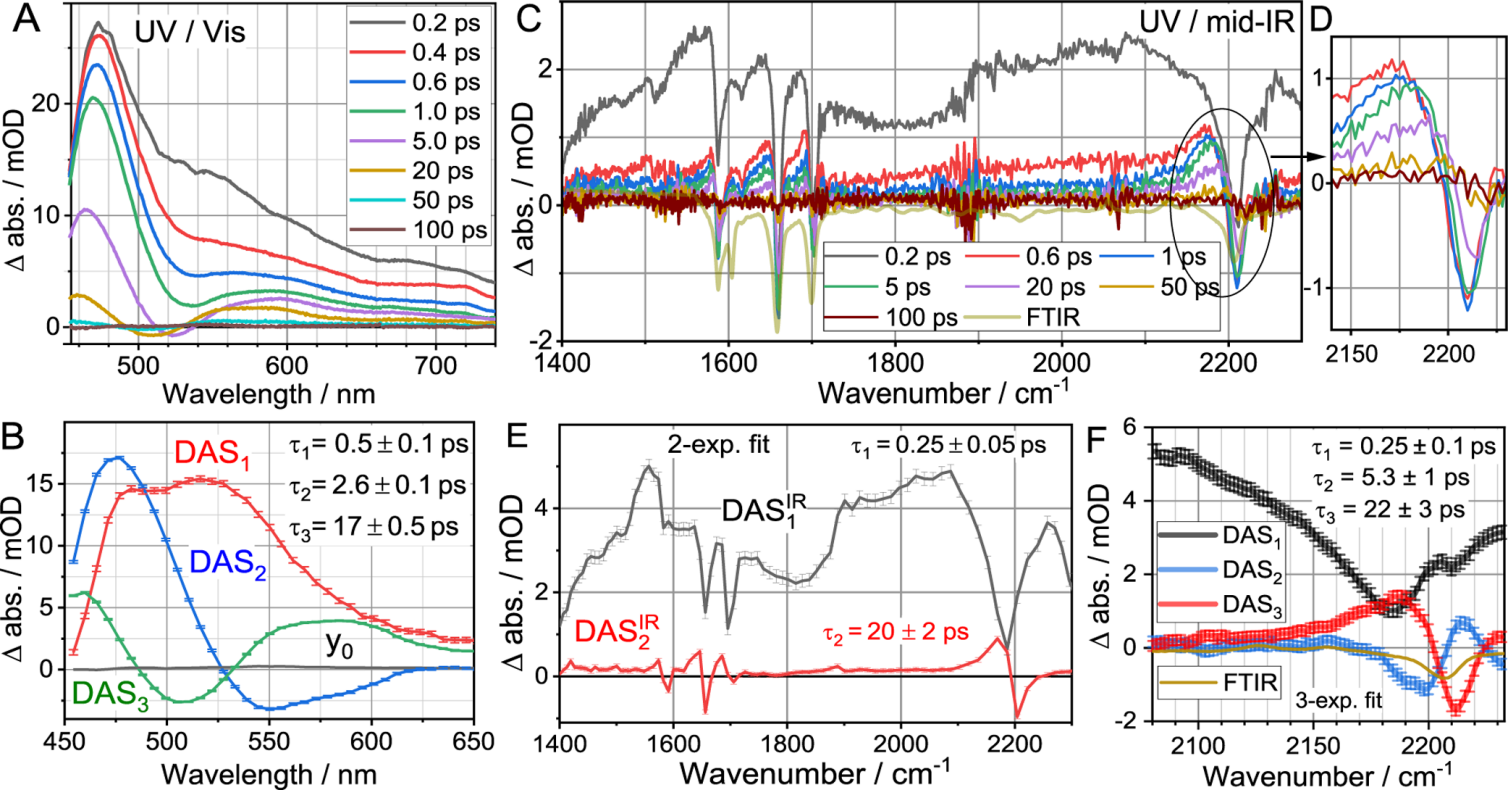
Transient spectra in the visible (A) and mid-IR (C, D) regions for Fc-C4-NAP in DCM measured at indicated time delays following 402 nm excitation. Panel C also shows a scaled linear absorption spectrum (FTIR). The results of the global analysis are shown in panels B (UV/vis) and E (UV/m-IR). A three-exponential function was used for the global analysis of the UV/vis data in the form of y=y0+DAS1(λ)exp(−tτ1)+DAS2(λ)exp(−tτ2)+DAS3(λ)exp(−tτ3) . A two-exponential function was used to fit the UV/mid-IR data in the whole region from 1400 to 2300 cm^–1^ (**E**) and a three-exponential function was used for the narrow spectral region around 2200 cm^–1^, as shown in panel **F**. The resulting characteristic times are shown in panels **B**, **E**, and **F** as insets.

#### 2.2.2 UV/mid-IR Results

Transient UV/mid-IR spectra measured over a broad range of frequencies from 1400 to 2250 cm^–1^ (Figure 3C) provide insight for assigning intermediate states. Surprisingly, the transient spectra measured at small delay times (0.2 and 0.6 ps) feature unusually broad absorption, spanning the entire detection region (Figure 3C). Such broad absorption cannot be caused by vibronic transitions, but rather is assigned to an electronic transition or several electronic transitions, similar to related findings in the literature.^69^ Note that no corresponding broad absorption was found in DMA-C4-NAP in DCM,^41^ suggesting involvement of the Fc states of Fc-C4-NAP. Featureless, broad mid-IR absorption bands have been previously reported for oxidized Pt­(II) acetylide complexes, attributed to acetylide-centered oxidation. In these systems, the CC ESA spans ∼1800–2000 cm^–1^ (fwhm ∼150 cm^–1^) with amplitudes smaller than those of carbonyl ESA.^70^ In contrast, in Fc–C4–NAP, the absorbance is significantly broader (1400–2300 cm^–1^) and comparable in amplitude to that of carbonyl ESA, indicating its electronic origin.

As the broad absorption is peaking at *t* = 0, the state from which the broad absorption originates is the bright NAP-based LE state. The broad absorption decays rapidly, with a time constant of ca. 0.25 ps (Figure 3C, E), indicating that the NAP-based LE state is short-lived. Surprisingly, spatially separated NAP-centered and Fc-centered states are coupled so well to result in such fast relaxation; TD-DFT analysis sheds light on the origin of these observations (see Section 3.3).

The global fit of the UV/mid-IR transient spectra involving the whole frequency range resulted in only two different DAS components, shown in Figure 3E. A three-exponential global analysis performed for the whole frequency range produced DAS components where two components are superimposable while differing by a sign and carrying essentially the same characteristic times, indicating that they are not independent. Note that the spectral changes in the 2200 cm^–1^ spectral region (Figure 3D) are clearly more complex than can be described by two exponential components, showing continuous blue shift of ESA peak apparent for *t* ≥ 5 ps, which results in a shift of the zero-crossing point. Such changes, however, are too small in amplitude to be identified in the global fit of the overall data set. To circumvent the difficulty, we performed a three-component global fit for the narrower spectral region, shown in Figure 3F.

The characteristic times obtained in the three-component fit are similar to those obtained for the global fit of the TA spectra in the visible (Figure 3A,B). The DAS spectrum and the characteristic time of the fast component are essentially the same as for the wide fit of Figure 3E. The third component has a similar shape to the slow component of Figure 3E with negative GSB and positive ESA parts. Such a shape of the DAS reports on the decay to zero of the whole spectrum as the positive portion of DAS_3_ matches the positive TA signal and thus reporting on its decay, while the negative portion of DAS_3_ matches the negative TA signal, also reporting on its decay to zero. Again, the DAS_3_ component reports on the disappearance of the TA signals with ca. 22 ps characteristic time. The middle component describes a shift of the transient spectrum to higher frequencies as its negative and positive parts both report an increase of the TA amplitudes and the increase is to the blue with respect to the features at earlier times. The characteristic time of DAS_2_ is ca. 5 ps, which seems to be too short for vibrational cooling (∼15 ps is expected) and could involve electronic relaxation as well as vibrational cooling. Because the characteristic time of the DAS_2_ component is rather short and there is a similar component in UV/vis spectral changes (Figure 3B), we assign it to electronic relaxation.

The ESA of ν_C≡C_ peaks at 2185 cm^–1^ (Figure 3F, red line), which is shifted by 22 cm^–1^ from that in the GS. So large frequency shift of the relatively long-lived state (ca. 20 ps) clearly indicates that the state is an electronically excited state, not a hot GS. In fact, formation of the hot GS is apparent at larger delays, most clearly seen in the spectra at *t* of 20 and 50 ps (Figure 3D), characterized by a small (<3 cm^–1^) ν_C≡C_ frequency red shift caused by small vibrational anharmonicities of ν_C≡C_ and other modes in the compound.^66–68^ This shift has not been captured by the three-component global fit, likely because of the small amplitudes of the changes and significant noise. A similarity of the characteristic time of electronic relaxation and vibrational relaxation makes the separation of different signal contributions more difficult.

While this long-lived state is clearly an electronic state, the ν_C≡C_ frequency shift of ca. 22 cm^–1^ is too small to assign the state as the CS state. A red shift of ca. 150 cm^–1^ was observed for ν_C≡C_ in DMA-C4-NAP where a nearly pure CS state is formed.^41,65,71–74^ Such strongly red-shifted absorption of ν_C≡C_ was not observed in Fc-C4-NAP (Figure 3E, red line). In addition, the IR intensity of its ν_C≡C_ ESA peak is only 1.3 times greater than that of the GS, compared to the 10-fold increase for the CS state in DMA-C4-NAP.^41^ The frequency shifts of the carbonyl modes (ν_ss_ and ν_as_) in the long-lived (∼20 ps) state are small (<3 cm^–1^, Figure 3C, E, F), which suggests that the long-lived state can have only a small contribution derived from the NAP moiety, thus indicating that it is predominantly Fc-based. TA measurements were also performed in toluene solvent. The characteristic times were found to be only slightly slower than those in DCM, caused by the lower polarity of toluene (see Section S7).

The experimental linear and nonlinear absorption data provide limiting insight for important questions regarding the nature of the peak at 500 nm in the linear absorption spectrum, the broad transient absorption in mid-IR, or the nature of the long-lived excited state with the 17–20 ps lifetime. The data also do not provide insight into how the states vary with the torsion angle, θ. TD-DFT analysis reveals a complex but trackable state pattern as a function of θ.

### 2.3 TD-DFT Computations

TD-DFT analysis was performed to characterize how the energies, dipole moments, and oscillator strengths of the 12 lowest-energy electronic states depend on θ. The energy difference for θ rotation in the GS was computed to be small, ca. 10 meV, which makes all θ angles thermally accessible at room temperature (Figure S3).

Figure 4A shows how the energies of the lowest 11 excited states depend on θ. The diameter of the circles in Figure 4A is proportional to the oscillator strength; Figure S16 shows the computed values of the oscillator strength directly. The lowest energy bright state of Fc-C4-NAP is expected to be the NAP-based LE state, observed at ca. 385 nm (Figure 2A). A large oscillator strength is found for state S7 at small θ angles and for state S8 at large θ angles (Figure 4A). Such complementary behavior as a function of θ can be interpreted as the coupling of two or more site states, producing their θ-dependent mixing. Because of the presence of orbitals that are fully delocalized across the compound (Figure 5A), the coupling pattern is complex for the S7 and S8 states and for many other states. Natural transition orbitals (NTO) show this complexity clearly (Figures S4–S14). For example, at small θ angles, state S7 is mostly NAP-based, although it involves the C4 bridge and partially involves Fc. As such, the S7 state has some charge transfer character, although its dipole moment is not very high at ca. 26D (Figure 4B). The state character at large θ values changes, and the state becomes essentially the CS state (Figure 5B), confirmed by its increased dipole moment of 33D compared to its dipole moment at small θ. Similar changes of the state character are found for S8 (Figure 5C, D), which at small θ angles has a large Fc → NAP CS contribution (53 + 24 = 77%, Figure 5C) as well as a Fc → Fc contribution of 21%. However, at large θ angles, the state becomes predominantly NAP-based, manifested in its small dipole moment (Figure 4B) and large oscillator strength (Figure 4A). Thus, the orbital delocalization results in efficient θ-dependent mixing of different localized states, so that the resulting state character changes drastically as a function of θ.

**Figure 4:**
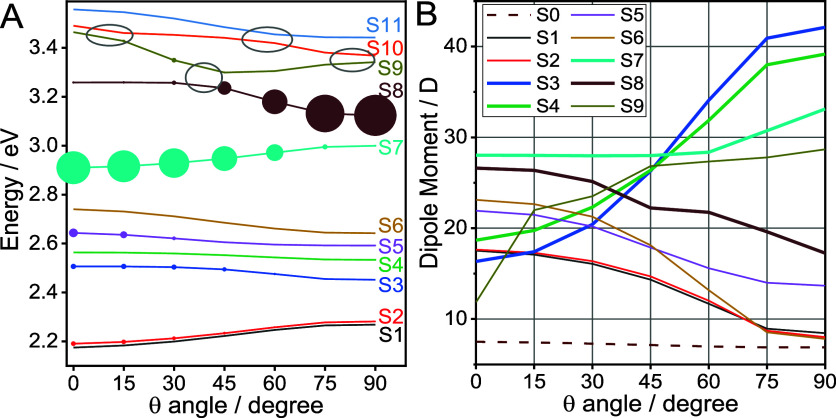
TD-DFT computed excited state energies (A) and permanent dipole moments (B) as a function of torsion angle, θ. The symbol size (diameter) in panel A represents the oscillator strength of the transition from the ground state.

**Figure 5:**
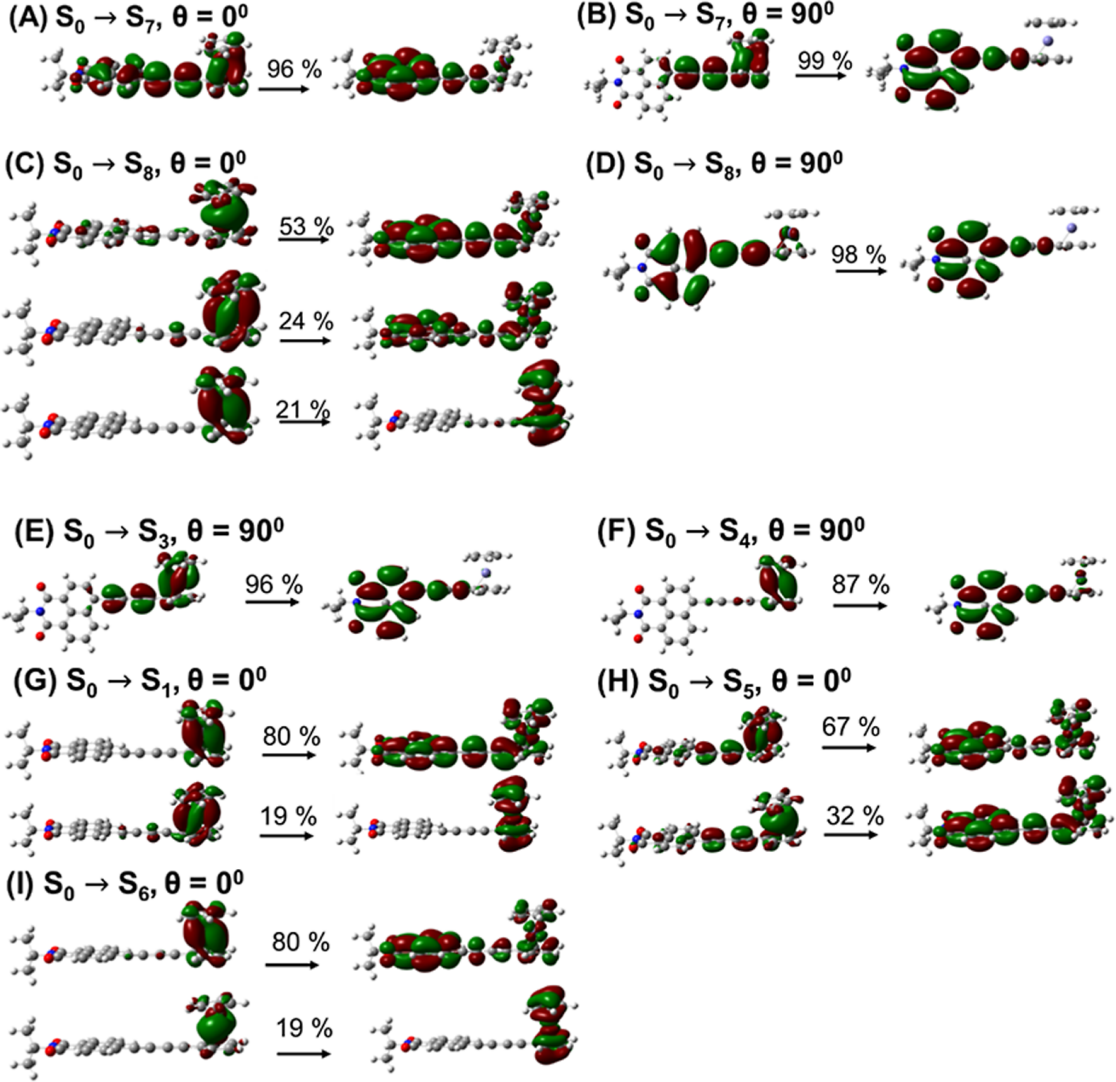
Natural transition orbitals (NTO) for the transitions to selected excited states from the ground state at selected θ angles (see more data in Figures S4–S14). The numbers above arrows indicate the contributions to the transition.

A CS state can be identified by its large dipole moment (Figure 4B). Such states at low energies are S3 and S4, featuring large dipole moments at θ = 90° of 43 and 39 D, respectively (Figure 4B), which corresponds approximately to a charge-separated state with a hole on Fc and an electron on NAP, Figures 5E,F, S5 and S6. Interestingly, there are two such states, supported by two orthogonally polarized orbitals of Fc (Figure 5G) with similar energies. State pairs S1, S2 and S5, S6 also feature orthogonally polarized hole states of Fc (see Figure 5H,I for states S5 and S6).

The TD-DFT analysis finds that the lowest energy excited states, S1 and S2, have very small oscillator strengths for all torsion angles (θ), and are localized primarily on the Fc moiety, associated with d-orbitals of Fe. Nevertheless, at small θ angles both states have Fc → NAP contributions (Figure 5G), which leads to higher dipole moments of ca. 17D, compared to 8D at θ = 90°, where such contributions were not found. The diabatic states’ coupling and mixing are apparent at higher energies as avoided crossings (S8–S11), labeled in Figure 4A with ovals. Oscillator strength sharing, as for example observed among states S8 and S9 at θ = 30°, is a clear indication of such coupling.

The TD-DFT computed linear absorption spectrum is shown in Figure S15. It describes fairly the low-frequency peak amplitudes, although the frequency of the NAP-based LE state was predicted to occur at a lower energy than that observed experimentally, likely caused by an overemphasis of orbital delocalization in the calculations.

The TD-DFT analysis describes the origin of the main experimental observations. The broad absorption in the 1400–2400 cm^–1^ spectral region (Figure 3C) is explained by the transition from states S7 and S8 to the states S9–S11. States S7–S8 and S9–S11 have components localized on the same moieties (Figures S11–S13), which increases the oscillator strength for these transitions. The light-induced transition from S7 to S8 may also account for some of the broad absorption in Fc-C4-NAP.

Figure 6 summarizes the processes that occur upon excitation of the S7/S8 bright states. Electronic relaxation to the S3/S4 states, which can be thought as two CS states, is not clearly observed as the states formed feature very small shifts of the ν_C≡C_ peak frequency compared to that in the GS. Therefore, we conclude that *k*_3_ ≪ *k*_1_ + *k*_2_. The S7/S8 state relaxation can populate S5/S6 and/or S1/S2 states. The fast decay of the broad absorption is assigned to these two processes together: *k*_1_ + *k*_2_ = (0.25 ps)^−1^. The observation of the mixed characters of states S7 and S8 makes it unsurprising that their relaxation rate is so fast. The existing data do not allow us to determine the relative contributions of these relaxation channels (*k*_1_ vs *k*_2_). The middle component observed in the UV/vis and UV/m-IR data, that is about 3–5 ps, can be assigned to either *k*_1_, *k*_2_, or *k*_4_. We concluded that this component is due to electronic relaxation or vibronic cooling rather than due to vibrational cooling. The vibrational cooling time is expected to be about 15 ps (*k*_6_), which approximately matches the electronic relaxation component of ca. 20 ps, attributed to S2/S1 → S0^hot^ relaxation (*k*_5_). This rate match results in a stronger influence of the two processes on one another, leading to an increased error in determining *k*_5_, the relaxation rate of the lowest energy excited state of Fc-C4-NAP to the GS.

**Figure 6:**
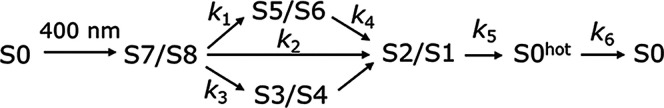
Summary of the processes occurring upon Fc-C4-NAP excitation by 402 nm pulses.

Interestingly, Fc-C4-NAP exhibits an additional higher-energy charge-separated state at ca. 330 nm. This state involves a transition from a different hole state of Fc, as revealed by the TD-DFT analysis.

The presence of Fe atom can potentially result in intersystem crossing (ISC) as fast as a few hundred femtoseconds, populating triplet excited states. Triplet state formation often results in longer lived ligand-localized states, as reported for Fe­(II) complexes,^75–77^ Pt­(II) donor–acceptor systems,^78–80^ and cobalt­(III) cyclam complexes.^59^ Notice, however, that relaxation of the initially excited NAP-based singlet state is significantly faster than a few hundred femtoseconds. There is no indication that an NAP-based triplet state is formed as a significant frequency shifts of ν_ss_ and ν_as_, expected in this case,^81–83^ were not observed with actual shifts at less than 3 cm^–1^. The lower energy Fc-centered states can undergo fast ISC. However, we do not have any evidence of such process, and the final excited states (S1/S2) remain short-lived (20 ps), suggesting that ISC may not play essential role in Fc-C4-NAP.

## 3 Concluding Remarks

The extent of charge separation in Fc-C4-NAP is influenced by the coupling of the pure LE and CS states, which was also observed in other DBA compounds, such as DMA-C4-NAP. Strong coupling, which occurs when the two pure states are in resonance, produces their strong mixing, leading to incomplete charge separation in each of the eigenstates. By tuning the energy of the pure CS state away from the LE state, such state mixing can be avoided, resulting in essentially pure CS eigenstates. The opportunity of manipulating the mixing motivated our selection of the electron donor, Fc, which is stronger than DMA, thus decreasing the energy of the pure CS state and ensuring, as expected, a higher polarization of the CS states. The Fc-C4-NAP compound, however, demonstrated complexity in the nature of its excited states, which was dictated by the presence of several dark excited states associated with the Fc moiety in the vicinity of the pure CS and LE states. Surprisingly, these Fc-localized states are found to be coupled strongly to both pure CS and LE states. The large coupling, estimated to be 200–500 cm^–1^ for different states pairs, and high density of Fc-based pure states, resulted in several strong coupling situations involving pure CS and LE states, occurring at different torsion angles (Figure 4A). The energies of pure LE and CS states vary significantly with the torsion angle, as for DMA-C4-NAP,^41^ while the energies of the Fc-based states do not. As a result, resonances with strong coupling cases occur at different θ values, linking pure LE and CS states to several pure Fc-based states. Some other pure Fc-based states, such as S1 and S2, are coupled weakly to the pure LE and CS states, nevertheless, leading to their wave function mixing, although the mixing is much smaller than in the case of S9–S11. Such mixing, strong and weak, results in fast energy relaxation from the bright, predominantly LE states, to the lowest energy excited states of Fc (S1 and S2), passing the CS eigenstates (S3–S4). The lifetime of the S1–S2 states is found to be ca. 20 ps.

The reason for the mixing of various diabatic states lies in the strength of their electronic coupling, which is mediated by the π-conjugated alkyne bridge and the high density of low-lying excited states associated with the Fc moiety. Such conditions were avoided in earlier DBA triads involving a Fc donor because of the use of saturated bridges, which greatly reduce the through-bond coupling, thus minimizing electronic communication between the donor and acceptor. The reduced communication results in slower charge separation but also avoids state mixing. For example, Albinsson and co-workers reported a diporphyrin system with a ferrocene donor linked via a nonconjugated bridge, where, despite the presence of low-lying Fc-based states, the charge-separated (CS) state was long-lived and exhibited little evidence of state mixing, suggesting weak coupling between the local and CS states.^84^ Similarly, Fukuzumi and colleagues studied Fc–Ph–C_60_ triads with alkyl linkers and observed efficient photoinduced electron transfer followed by formation of long-lived CS states, again consistent with weak donor–acceptor coupling arising from spatial separation.^85^ In another study, Imahori et al. found that in Fc–oligophenylene–C_60_ systems, improved conjugation slightly enhanced electronic communication, but state mixing remained minimal.^86^ Taken together, these examples show how the combination of a conjugated bridge and the electronic structure of the Fc donor in our system creates the conditions needed to couple diabatic excited states. Fast energy relaxation between excited ZnTPP and Fc directly tethered to one of the phenyl moieties of ZnTPP was reported by Wasielewski and co-workers, indicating dominance of the energy transfer to low-laying Fc states compared to the CS process.^64^

Experimental observations of this study, combined with TD-DFT analysis, offer a clear picture of the excited-state characters and dynamics in Fc-C4-NAP. Our findings demonstrate how the structural flexibility offered by the alkyne bridge and Fc-based dark diabatic excited states produce a distinctly different excited states compared to DBA species with simpler, planar, fully organic donor moieties. The findings underscore the utility of incorporating both electronic and conformational factors to understand and to control the photoinduced electron transfer in donor–bridge–acceptor systems. The insights gained provide guidance toward formulating design principles for ultrafast charge separation modules of utility in molecular materials for solar energy, photocatalysis, and optoelectronic applications.

## Supplementary Material



## Notes

1H NMR Spectra of Fc-C4-NAPiPr. The peak labels are shown in inset (PDF)

## References

[1] Duncan T. V., Ghoroghchian P. P., Rubtsov I. V., Hammer D. A., Therien M. J. (2008). Ultrafast
excited-state dynamics of nanoscale near-infrared emissive polymersomes. J. Am. Chem. Soc..

[2] Shan B., Nayak A., Williams O. F., Yost D. C., Polizzi N. F., Liu Y., Zhou N., Kanai Y., Moran A. M., Therien M. J. (2019). Excitation energy-dependent photocurrent switching in a single-molecule
photodiode. Proc. Natl. Acad. Sci. U. S. A..

[3] Shi Y., Frattarelli D., Watanabe N., Facchetti A., Cariati E., Righetto S., Tordin E., Zuccaccia C., Macchioni A., Wegener S. L. (2015). Ultra-high-response,
multiply twisted electro-optic chromophores: influence of π-system
elongation and interplanar torsion on hyperpolarizability. J. Am. Chem. Soc..

[4] Bai Y., Rawson J., Roget S. A., Olivier J.-H., Lin J., Zhang P., Beratan D. N., Therien M. J. (2017). Controlling the
excited-state dynamics of low band gap, near-infrared absorbers via
proquinoidal unit electronic structural modulation. Chem. Sci..

[5] Fenenko L., Shao G., Orita A., Yahiro M., Otera J., Svechnikov S., Adachi C. (2007). Electrical properties of 1, 4-bis
(4-(phenylethynyl) phenylethynyl) benzene and its application for
organic light emitting diodes. Chem. Commun..

[6] Nayak A., Park J., De Mey K., Hu X., Duncan T. V., Beratan D. N., Clays K., Therien M. J. (2016). Large hyperpolarizabilities
at telecommunication-relevant wavelengths in donor–acceptor–donor
nonlinear optical chromophores. ACS Cent. Sci..

[7] Jovaišaitė J., Baronas P., Jonusauskas G., Gudeika D., Gruodis A., Gražulevičius J. V., Juršėnas S. (2023). TICT compounds
by design: comparison of two naphthalimide-π-dimethylaniline
conjugates of different lengths and ground state geometries. Phys. Chem. Chem. Phys..

[8] Dumur F., Gautier N., Gallego-Planas N., Şahin Y., Levillain E., Mercier N., Hudhomme P., Masino M., Girlando A., Lloveras V. (2004). Novel fused D–
A dyad and A– D– A triad incorporating tetrathiafulvalene
and p-benzoquinone. J. Org. Chem..

[9] Heitzer H. M., Marks T. J., Ratner M. A. (2015). Molecular
Donor–Bridge–Acceptor
Strategies for High-Capacitance Organic Dielectric Materials. J. Am. Chem. Soc..

[10] Kandhadi J., Yeduru V., Bangal P. R., Giribabu L. (2015). Corrole–ferrocene
and corrole–anthraquinone dyads: synthesis, spectroscopy and
photochemistry. Phys. Chem. Chem. Phys..

[11] Kirketerp M. B. S., Petersen M. Å., Wanko M., Zettergren H., Rubio A., Nielsen M. B., Nielsen S. B. (2010). Double-Bond versus
Triple-Bond Bridges: Does it Matter for the Charge-Transfer Absorption
by Donor–Acceptor Chromophores?. ChemPhysChem.

[12] Van
Dyck C., Ratner M. A. (2015). Molecular rectifiers: a new design based on asymmetric
anchoring moieties. Nano Lett..

[13] Yamazaki Y., Umemoto A., Ishitani O. (2016). Photochemical
Hydrogenation of π-Conjugated
Bridging Ligands in Photofunctional Multinuclear Complexes. Inorg. Chem..

[14] Ricks A. B., Solomon G. C., Colvin M. T., Scott A. M., Chen K., Ratner M. A., Wasielewski M. R. (2010). Controlling
Electron Transfer in
Donor–Bridge–Acceptor Molecules Using Cross-Conjugated
Bridges. J. Am. Chem. Soc..

[15] Dickson-Karn N. M., Olson C. M., Leu W. C., Hartley C. S. (2014). Intramolecular charge
transfer in donor-bridge-acceptor compounds with paired linearly conjugated
or cross-conjugated pathways. J. Phys. Org.
Chem..

[16] Hoffman D. P., Lee O. P., Millstone J. E., Chen M. S., Su T. A., Creelman M., Fréchet J. M., Mathies R. A. (2013). Electron transfer
dynamics of triphenylamine dyes bound to TiO2 nanoparticles from femtosecond
stimulated Raman spectroscopy. J. Phys. Chem.
C.

[17] Ricks A. B., Brown K. E., Wenninger M., Karlen S. D., Berlin Y. A., Co D. T., Wasielewski M. R. (2012). Exponential
Distance Dependence of
Photoinitiated Stepwise Electron Transfer in Donor–Bridge–Acceptor
Molecules: Implications for Wirelike Behavior. J. Am. Chem. Soc..

[18] Priyadarshy S., Therien M. J., Beratan D. N. (1996). Acetylenyl-linked, porphyrin-bridged,
donor– acceptor molecules: a theoretical analysis of the molecular
first hyperpolarizability in highly conjugated push– pull chromophore
structures. J. Am. Chem. Soc..

[19] Wang R., Brugh A. M., Rawson J., Therien M. J., Forbes M. D. (2017). Alkyne-bridged
multi [copper (ii) porphyrin] structures: Nuances of orbital symmetry
in long-range, through-bond mediated, isotropic spin exchange interactions. J. Am. Chem. Soc..

[20] Smith M. E., Flynn E. L., Fox M. A., Trottier A., Wrede E., Yufit D. S., Howard J. A., Ronayne K. L., Towrie M., Parker A. W. (2008). Facile photoinduced charge separation through
a cyanoacetylide bridge in a heterobimetallic Fe (ii)–Re (i)
complex. Chem. Commun..

[21] Dereka B., Balanikas E., Rosspeintner A., Li Z., Liska R., Vauthey E. (2024). Excited-State Symmetry Breaking and Localization in
a Noncentrosymmetric Electron Donor–Acceptor–Donor Molecule. J. Phys. Chem. Lett..

[22] Rizzuto F. J., Hua C., Chan B., Faust T. B., Rawal A., Leong C. F., Hook J. M., Kepert C. J., D’Alessandro D. M. (2015). The electronic,
optical and magnetic consequences of delocalization in multifunctional
donor–acceptor organic polymers. Phys.
Chem. Chem. Phys..

[23] Van
Dyck C., Marks T. J., Ratner M. A. (2017). Chain Length Dependence of the Dielectric
Constant and Polarizability in Conjugated Organic Thin Films. ACS Nano.

[24] Kivala M., Diederich F. (2009). Acetylene-derived strong organic acceptors for planar
and nonplanar push– pull chromophores. Acc. Chem. Res..

[25] Hoffmann M., Kärnbratt J., Chang M. H., Herz L. M., Albinsson B., Anderson H. L. (2008). Enhanced π conjugation around a porphyrin [6]
nanoring. Angew. Chem., Int. Ed..

[26] Leu W. C., Fritz A. E., Digianantonio K. M., Hartley C. S. (2012). Push–pull
macrocycles: donor–acceptor compounds with paired linearly
conjugated or cross-conjugated pathways. J.
Org. Chem..

[27] Leu W. C., Hartley C. S. (2013). A push–pull
macrocycle with both linearly conjugated
and cross-conjugated bridges. Org. Lett..

[28] Nilsson D., Watcharinyanon S., Eng M., Li L., Moons E., Johansson L. S., Zharnikov M., Shaporenko A., Albinsson B., Mårtensson J. (2007). Characterization of self-assembled
monolayers of oligo (phenyleneethynylene) derivatives of varying shapes
on gold: Effect of laterally extended π-systems. Langmuir.

[29] Heckmann A., Lambert C. (2012). Organic mixed-valence compounds: a playground for electrons
and holes. Angew. Chem., Int. Ed..

[30] Göransson E., Emanuelsson R., Jorner K., Markle T. F., Hammarström L., Ottosson H. (2013). Charge transfer through cross-hyperconjugated versus
cross-π-conjugated bridges: an intervalence charge transfer
study. Chem. Sci..

[31] Winters M. U., Kärnbratt J., Eng M., Wilson C. J., Anderson H. L., Albinsson B. (2007). Photophysics of a butadiyne-linked porphyrin dimer:
influence of conformational flexibility in the ground and first singlet
excited state. J. Phys. Chem. C.

[32] Beratan D. N., Skourtis S. S., Balabin I. A., Balaeff A., Keinan S., Venkatramani R., Xiao D. (2009). Steering Electrons on Moving Pathways. Acc.
Chem. Res..

[33] Skourtis S.
S., Waldeck D. H., Beratan D. N. (2010). Fluctuations in Biological and Bioinspired
Electron-Transfer Reactions. Annu. Rev. Phys.
Chem..

[34] Beratan D. N., Liu C., Migliore A., Polizzi N. F., Skourtis S. S., Zhang P., Zhang Y. (2015). Charge Transfer
in Dynamical Biosystems, or The Treachery of (Static)
Images. Acc. Chem. Res..

[35] Jiang F., Trupp D. I., Algethami N., Zheng H., He W., Alqorashi A., Zhu C., Tang C., Li R., Liu J. (2019). Turning the Tap: Conformational control of quantum
interference to modulate single-molecule conductance. Angew. Chem..

[36] Lin Z., Lawrence C. M., Xiao D., Kireev V. V., Skourtis S. S., Sessler J. L., Beratan D. N., Rubtsov I. V. (2009). Modulating Unimolecular
Charge Transfer by Exciting Bridge Vibrations. J. Am. Chem. Soc..

[37] Yue Y., Grusenmeyer T., Ma Z., Zhang P., Schmehl R. H., Beratan D. N., Rubtsov I. V. (2015). Electron transfer rate modulation
in a compact Re­(i) donor–acceptor complex. Dalton Trans..

[38] Ma Z., Lin Z., Lawrence C. M., Rubtsov I. V., Antoniou P., Skourtis S. S., Zhang P., Beratan D. N. (2018). How can infra-red excitation both
accelerate and slow charge transfer in the same molecule?. Chem. Sci..

[39] Rubtsov I. V., Kang Y. K., Redmore N. P., Allen R. M., Zheng J., Beratan D. N., Therien M. J. (2004). The Degree of Charge Transfer in
Ground and Charge-Separated States Revealed by Ultrafast Visible Pump/Mid-IR
Probe Spectroscopy. J. Am. Chem. Soc..

[40] Yue Y., Grusenmeyer T., Ma Z., Zhang P., Schmehl R. H., Beratan D. N., Rubtsov I. V. (2014). Full-Electron
Ligand-to-Ligand Charge
Transfer in a Compact Re­(I) Complex. J. Phys.
Chem. A.

[41] Li X., Valdiviezo J., Banziger S. D., Zhang P., Ren T., Beratan D. N., Rubtsov I. V. (2020). Symmetry controlled photo-selection
and charge separation in butadiyne-bridged donor–bridge–acceptor
compounds. Phys. Chem. Chem. Phys..

[42] Wu K.-Q., Guo J., Yan J.-F., Xie L.-L., Xu F.-B., Bai S., Nockemann P., Yuan Y.-F. (2012). Ruthenium (II) bis (terpyridine)
electron transfer complexes with alkynyl–ferrocenyl bridges:
synthesis, structures, and electrochemical and spectroscopic studies. Dalton Trans..

[43] Yadav I. S., Kaswan R. R., Liyanage A., Misra R., D’Souza F. (2024). Dimethylaniline-Tetracyanobutadiene
and Dimethylaniline-Extended-Tetracyanobutadiene Functionalized BODIPYs
Witnessing Ultrafast Charge Transfer. J. Phys.
Chem. C.

[44] Kealy T. J., Pauson P. L. (1951). A New Type of Organo-Iron Compound. Nature.

[45] Islam S., Wang F. (2015). The d-electrons of Fe in ferrocene: the excess orbital energy spectrum
(EOES). RSC Adv..

[46] Yamaguchi Y., Kutal C. (1999). Efficient photodissociation of anions from benzoyl-functionalized
ferrocene complexes. Inorg. Chem..

[47] Gagne R. R., Koval C. A., Lisensky G. C. (1980). Ferrocene
as an internal standard
for electrochemical measurements. Inorg. Chem..

[48] Wasielewski M. R. (2006). Energy,
charge, and spin transport in molecules and self-assembled nanostructures
inspired by photosynthesis. J. Org. Chem..

[49] Le T. H., Nguyen V. Q., Trippe-Allard G., Lacroix J.-C., Martin P. (2020). Dithienylpyrrole
Electrografting on a Surface through the Electroreduction of Diazonium
Salts. Electrochem.

[50] Peiris C. R., Ciampi S., Dief E. M., Zhang J., Canfield P. J., Le Brun A. P., Kosov D. S., Reimers J. R., Darwish N. (2020). Spontaneous
S–Si bonding of alkanethiols to Si (111)–H: towards
Si–molecule–Si circuits. Chem.
Sci..

[51] Li T., Dief E. M., Lyu X., Rahpeima S., Ciampi S., Darwish N. (2021). Nanoscale Silicon Oxide
Reduces Electron Transfer Kinetics
of Surface-Bound Ferrocene Monolayers on Silicon. J. Phys. Chem. C.

[52] Bejarano F., Gutiérrez D., Catalán-Toledo J., Roca-Sanjuán D., Gierschner J., Veciana J., Mas-Torrent M., Rovira C., Crivillers N. (2022). Photoswitching activation of a ferrocenyl-stilbene
analogue by its covalent grafting to gold. Phys.
Chem. Chem. Phys..

[53] Gupta N. K., Schultz T., Karuppannan S. K., Vilan A., Koch N., Nijhuis C. A. (2021). The energy level alignment of the ferrocene–EGaIn
interface studied with photoelectron spectroscopy. Phys. Chem. Chem. Phys..

[54] Braga S. S., Silva A. M. (2013). A new age for iron: antitumoral ferrocenes. Organometallics.

[55] Pal A., Ranjan Bhatta S., Thakur A. (2021). Recent advances in the development
of ferrocene based electroactive small molecules for cation recognition:
A comprehensive review of the years 2010–2020. Coord. Chem. Rev..

[56] Ghosh A., Mishra S., Giri S., Mobin S. M., Bera A., Chatterjee S. (2018). Electrolyte-free
dye-sensitized solar cell with high
open circuit voltage using a bifunctional ferrocene-based cyanovinyl
molecule as dye and redox couple. Organometallics.

[57] Liu J.-J., Li N., Sun J.-W., Liu J., Dong L.-Z., Yao S.-J., Zhang L., Xin Z.-F., Shi J.-W., Wang J.-X. (2021). Ferrocene-functionalized
polyoxo-titanium cluster for CO2 photoreduction. ACS Catal..

[58] Siemsen P., Livingston R. C., Diederich F. (2000). Acetylenic coupling: a powerful tool
in molecular construction. Angew. Chem., Int.
Ed..

[59] Banziger S. D., Li X., Valdiviezo J., Zeller M., Zhang P., Beratan D. N., Rubtsov I. V., Ren T. (2019). Unsymmetrical Bis-Alkynyl Complexes
Based on Co (III)­(cyclam): Synthesis, Ultrafast Charge Separation,
and Analysis. Inorg. Chem..

[60] Salzner U. (2013). Quantitatively
Correct UV-vis Spectrum of Ferrocene with TDB3LYP. J. Chem. Theory Comput..

[61] Labulo A. H., Omondi B., Nyamori V. O. (2019). Synthesis, crystal structures and
electrochemical properties of ferrocenyl imidazole derivatives. Heliyon.

[62] Burke J. H., Bae D. Y., Wallick R. F., Dykstra C. P., Rossi T. C., Smith L. E., Leahy C. A., Schaller R. D., Mirica L. M., Vura-Weis J., van der Veen R. M. (2024). High-Spin
State of a Ferrocene Electron
Donor Revealed by Optical and X-ray Transient Absorption Spectroscopy. J. Am. Chem. Soc..

[63] Mendis, K. C. L. ; Xiao, V. ; Jesús, C. ; Susannah, D. ; Zhang, P. ; Ren, T. ; Beratan, D. N. ; Rubtsov, I. V. Photo-induced electron transfer dynamics and its mid-IR modulation of an ethyne bridged donor-acceptor complex, Phys. Chem. Chem. Phys. 2025 27, 20668 20679 10.1039/D5CP02004B. 40955433

[64] Poddutoori P., Co D. T., Samuel A. P. S., Kim C. H., Vagnini M. T., Wasielewski M. R. (2011). Photoinitiated multistep charge separation in ferrocene–zinc
porphyrin–diiron hydrogenase model complex triads. Energy Environ. Sci..

[65] Mendis K. C., Li X., Valdiviezo J., Banziger S. D., Zhang P., Ren T., Beratan D. N., Rubtsov I. V. (2024). Electron transfer rate modulation
with mid-IR in butadiyne-bridged donor–bridge–acceptor
compounds. Phys. Chem. Chem. Phys..

[66] Rubtsova N. I., Rubtsov I. V. (2015). Vibrational energy
transport in molecules studied by
relaxation-assisted two-dimensional infrared spectroscopy. Annu. Rev. Phys. Chem..

[67] Lin Z., Keiffer P., Rubtsov I. V. (2011). A Method
for Determining Small Anharmonicity
Values from 2DIR Spectra Using Thermally Induced Shifts of Frequencies
of High-Frequency Modes. J. Phys. Chem. B.

[68] Rubtsov I. V., Burin A. L. (2019). Ballistic and diffusive
vibrational energy transport
in molecules. J. Chem. Phys..

[69] Yang W., Liu Y., Edvinsson T., Castner A., Wang S., He S., Ott S., Hammarstrom L., Lian T. (2021). Photoinduced Fano resonances between
quantum confined nanocrystals and adsorbed molecular catalysts. Nano Lett..

[70] Archer S. A., Keane T., Delor M., Bevon E., Auty A. J., Chekulaev D., Sazanovich I. V., Towrie M., Meijer A. J. H. M., Weinstein J. A. (2017). Directly
Coupled Versus Spectator Linkers on Diimine
PtII Acetylides Change the Structure, Keep the Function?. Chemistry–A European Journal.

[71] Fureraj I., Wega J., Balanikas E., Puji Pamungkas K. K., Sakai N., Matile S., Vauthey E. (2024). Excitation-Wavelength-Dependent
Photophysics of a Torsionally Disordered Push–Pull Dye. J. Phys. Chem. Lett..

[72] Govind C., Balanikas E., Sanil G., Gryko D. T., Vauthey E. (2024). Structural
and solvent modulation of symmetry-breaking charge-transfer pathways
in molecular triads. Chem. Sci..

[73] Dereka B., Svechkarev D., Rosspeintner A., Tromayer M., Liska R., Mohs A. M., Vauthey E. (2017). Direct observation of a photochemical
alkyne–allene reaction and of a twisted and rehybridized intramolecular
charge-transfer state in a donor–acceptor dyad. J. Am. Chem. Soc..

[74] Scattergood P. A., Delor M., Sazanovich I. V., Bouganov O. V., Tikhomirov S. A., Stasheuski A. S., Parker A. W., Greetham G. M., Towrie M., Davies E. S., Meijer A. J., Weinstein J. A. (2014). Electron
transfer dynamics and excited state branching in a charge-transfer
platinum­(II) donor-bridge-acceptor assembly. Dalton Trans..

[75] Gawelda W., Cannizzo A., Pham V.-T., van Mourik F., Bressler C., Chergui M. (2007). Ultrafast Nonadiabatic Dynamics of
[FeII­(bpy)­3]­2+ in Solution. J. Am. Chem. Soc..

[76] Lindh L., Pascher T., Persson S., Goriya Y., Wärnmark K., Uhlig J., Chábera P., Persson P., Yartsev A. (2023). Multifaceted
Deactivation Dynamics of Fe­(II) N-Heterocyclic Carbene Photosensitizers. J. Phys. Chem. A.

[77] Cebrián C., Pastore M., Monari A., Assfeld X., Gros P. C., Haacke S. (2022). Ultrafast Spectroscopy of Fe­(II)
Complexes Designed
for Solar-Energy Conversion: Current Status and Open Questions. ChemPhysChem.

[78] Farrow G. A., Quick M., Kovalenko S. A., Wu G., Sadler A., Chekulaev D., Chauvet A. A. P., Weinstein J. A., Ernsting N. P. (2021). On the intersystem crossing rate in a Platinum­(ii)
donor–bridge–acceptor triad. Phys.
Chem. Chem. Phys..

[79] Whittle C. E., Weinstein J. A., George M. W., Schanze K. S. (2001). Photophysics of
Diimine Platinum­(II) Bis-Acetylide Complexes. Inorg. Chem..

[80] Liu L., Huang D., Draper S. M., Yi X., Wu W., Zhao J. (2013). Visible light-harvesting trans bis­(alkylphosphine)
platinum­(II)-alkynyl
complexes showing long-lived triplet excited states as triplet photosensitizers
for triplet-triplet annihilation upconversion. Dalton Trans..

[81] Delor M., Keane T., Scattergood P. A., Sazanovich I. V., Greetham G. M., Towrie M., Meijer A. J., Weinstein J. A. (2015). On the
mechanism of vibrational control of light-induced charge transfer
in donor–bridge–acceptor assemblies. Nat. Chem..

[82] Sykes D., Parker S. C., Sazanovich I. V., Stephenson A., Weinstein J. A., Ward M. D. (2013). d→ f Energy transfer in Ir
(III)/Eu (III) dyads: Use of a naphthyl spacer as a spatial and energetic
“stepping stone”. Inorg. Chem..

[83] Ivalo I. I., Wu G., Cowin R. A., Scattergood P. A., Cheng T., Shipp J. D., Sazanovich I. V., Chekulaev D., Weinstein J. A. (2025). Effect
of a peripheral substituent on the ultrafast dynamics and the rate
of intersystem crossing in the charge-transfer states of platinum
(ii) acetylides. Phys. Chem. Chem. Phys..

[84] Winters M. U., Kärnbratt J., Eng M., Wilson C. J., Anderson H. L., Albinsson B. (2007). Photophysics
of a Butadiyne-Linked Porphyrin Dimer:
Influence of Conformational Flexibility in the Ground and First Singlet
Excited State. J. Phys. Chem. C.

[85] Fukuzumi S., Ohkubo K., Suenobu T. (2014). Long-Lived
Charge Separation and
Applications in Artificial Photosynthesis. Acc.
Chem. Res..

[86] Imahori H., Guldi D. M., Tamaki K., Yoshida Y., Luo C., Sakata Y., Fukuzumi S. (2001). Charge Separation
in a Novel Artificial
Photosynthetic Reaction Center Lives 380 ms. J. Am. Chem. Soc..

