# Supplementary material for: Ultrafast Electron Dynamics of a Ferrocene-Based Butadiyne-Bridged Complex
Source: J Phys Chem A. 2026 Mar 16;130(12):2575–84. doi: 10.1021/acs.jpca.5c08653 (PMC13034416; doi:10.1021/acs.jpca.5c08653)
Supplement: Supplementary file 1 [file jp5c08653_si_001.pdf]

Supporting Information

Ultrafast Electron Dynamics of a Ferrocene-Based Butadiyne-Bridged Complex

Kasun C. Mendis,[a*] Jesús Valdiviezo,[b,c] Susannah D. Cox,[f] Peng Zhang,[b] Xiao Li,[a] Tong Ren,[f] David N. Beratan,[b,d,e*] and Igor V. Rubtsov[a*]

[a] *Department of Chemistry, Tulane University, New Orleans, LA 70118, USA*
[b] *Department of Chemistry, Duke University, Durham, North Carolina 27708, USA*
[c] *Seccion Quimica, Departamento de Ciencias, Pontificia Universidad Catolica del Peru, San Miguel, Lima 15088, Peru*
[d] *Department of Physics, Duke University, Durham, North Carolina 27708, USA*
[e] *Department of Biochemistry, Duke University, Durham, North Carolina 27710, USA*
[f] *Department of Chemistry, Purdue University, West Lafayette, Indiana 47907, USA*

Table of Content

S1. Details of synthesis……………………………………………….....………..……………………….S2

S2. ^{1}H NMR and ^{13}C NMR spectra…………………………………………………………………………..S3

S3. DFT computed ground state energies………………………………………………………………..S4

S4. TD-DFT computed natural transition orbital analysis…………………………….…..………………..S5

S5. TD-DFT modeled linear absorption spectrum……....……………………..……………………………..S16

S6. Variation of oscillator strength with torsion angle……...……………………..………………… ………S17

S7. Photoinduced dynamics of Fc-C4NAP in toluene……………………………………………..……..S17

* To whom correspondence should be addressed. E-mail: dmendis@tulane.edu, david.beratan@duke.edu, irubtsov@tulane.edu

S1. Details of synthesis

1.1. Materials and measurements

4-bromo-ethynyl-N-isopropyl-1,8-naphthalimide (BrC_2NAP^{iPr}) was prepared according to literature procedure [1]. THF was distilled over Na/benzophenone under a N_2 atmosphere. CH_2Cl_2 was distilled over CaH_2 under N_2. iPr_2NH was distilled over NaOH under a N_2 atmosphere. Ethynyl ferrocene was also prepared according to literature procedure was also synthesized according to literature procedure [2]. Pyridine was purchased from Mallinckrodt Chemicals and dried over hot sieves. All reactions were carried out using Schlenk techniques under N_2. UV-Vis-NIR spectra were obtained with a JASCO V-670 UV-Vis-NIR spectrophotometer. Infrared spectra were obtained on a JASCO FT-IR 6300 spectrometer via ATR on a diamond crystal. Emission spectra were measured on a Varian Cary Eclipse fluorescence spectrophotometer. ^{1}H NMR spectra were recorded on a Varian MERCURY300 NMR and a Varian Inova300.

[1] Li, X.; Valdiviezo, J.; Banziger, S. D.; Zhang, P.; Ren, T.; Beratan, D. N.; Rubtsov, I. V. *Phys. Chem. Chem. Phys.*, *22* (2020), 9664-9676

[2] G. Doisneau, G. Balavoine, T. Fillebeen-Khan, *J. Organomet. Chem.*, 425 (1992) 113-117.

Synthesis: (0.6gram scale) A 3-kneck round bottom flask was fitted with a side-arm containing CuI (741mg, 3.88 mmol) and charged with ethynylferrocene (764mg, 3.78 mmol) and dry THF (10 mL). Upon cooling to -10°C, nBuLi (2.5 M in hexane, 1.5 mL, 3.75 mmol) was added while stirring and warming to 0°C, the solution was dark red. After 20 min, CuI was added and the solution immediately turned dark brown. It was allowed to stir and warm to room temperature for 1hr. Pyridine (20 mL) was added while the solution stirred at 0°C. A solution of dry THF (30 mL) and BrC_2NAP^{iPr} (626mg, 1.88 mmol) was added dropwise over 1 h, the solution turned dark red. Upon complete addition, the reaction was allowed to stir an additional 10 min and quenched with wet THF. The solvent was removed under vacuum. The crude material was extracted from brine with CH_2Cl_2. Purification of the vibrant red solution was achieved through column chromatography (silica gel, 1:4 Hexane:CH_2Cl_2) yielded 118 mg of **Fc-C_4-NAP^{iPr}** as a dark purple powder and orange crystalline solid. (14% based on BrC_2NAP^{iPr}). ^{1}H NMR (300 MHz, Chloroform-*d*) δ 8.69 – 8.56 (m, 2H), 8.50 (d, *J* = 7.7 Hz, 1H), 7.93 (d, *J* = 7.7 Hz, 1H), 7.82 (dd, *J* = 8.3, 7.3 Hz, 1H), 5.43 (dt, *J* = 13.9, 6.9 Hz, 1H), 4.60 (t, *J* = 1.9 Hz, 2H), 4.34 (t, *J* = 1.9 Hz, 2H), 4.31 (s, 5H), 1.60 (d, *J* = 6.9 Hz, 6H). Visible spectra, λ_{max} (nm, ε (M^{-1} cm^{-1})): 278 (14,400), 383 (31,000), 490 (5,110); IR (cm^{-1}): C=O: 1660 (s), 1700 (s); C≡C: 2200 (s).

S2. ^{1}H NMR and ^{13}C NMR spectra

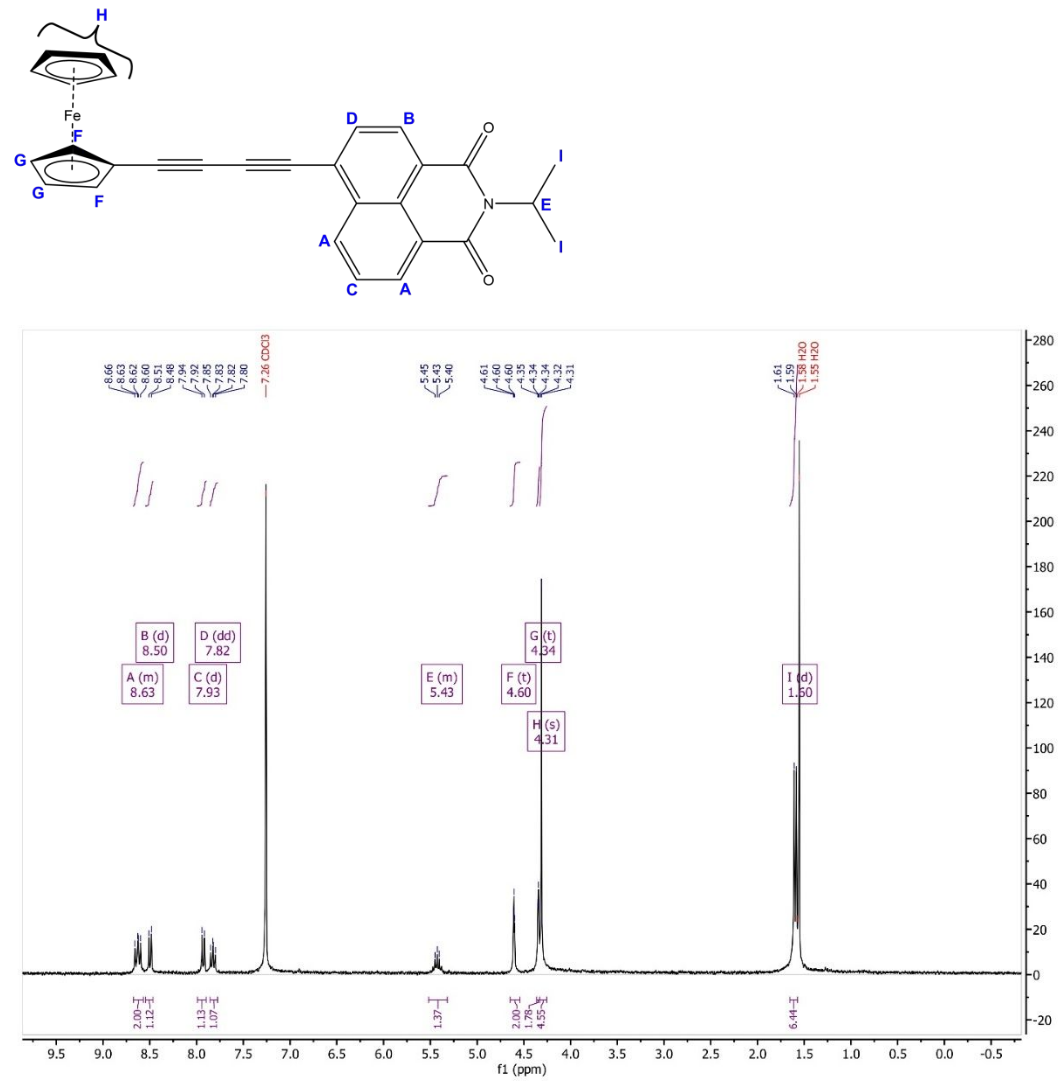

Figure S1. ^{1}H NMR Spectra of **Fc-C$_4$-NAP^{iPr}**. The peak labels are shown in inset.

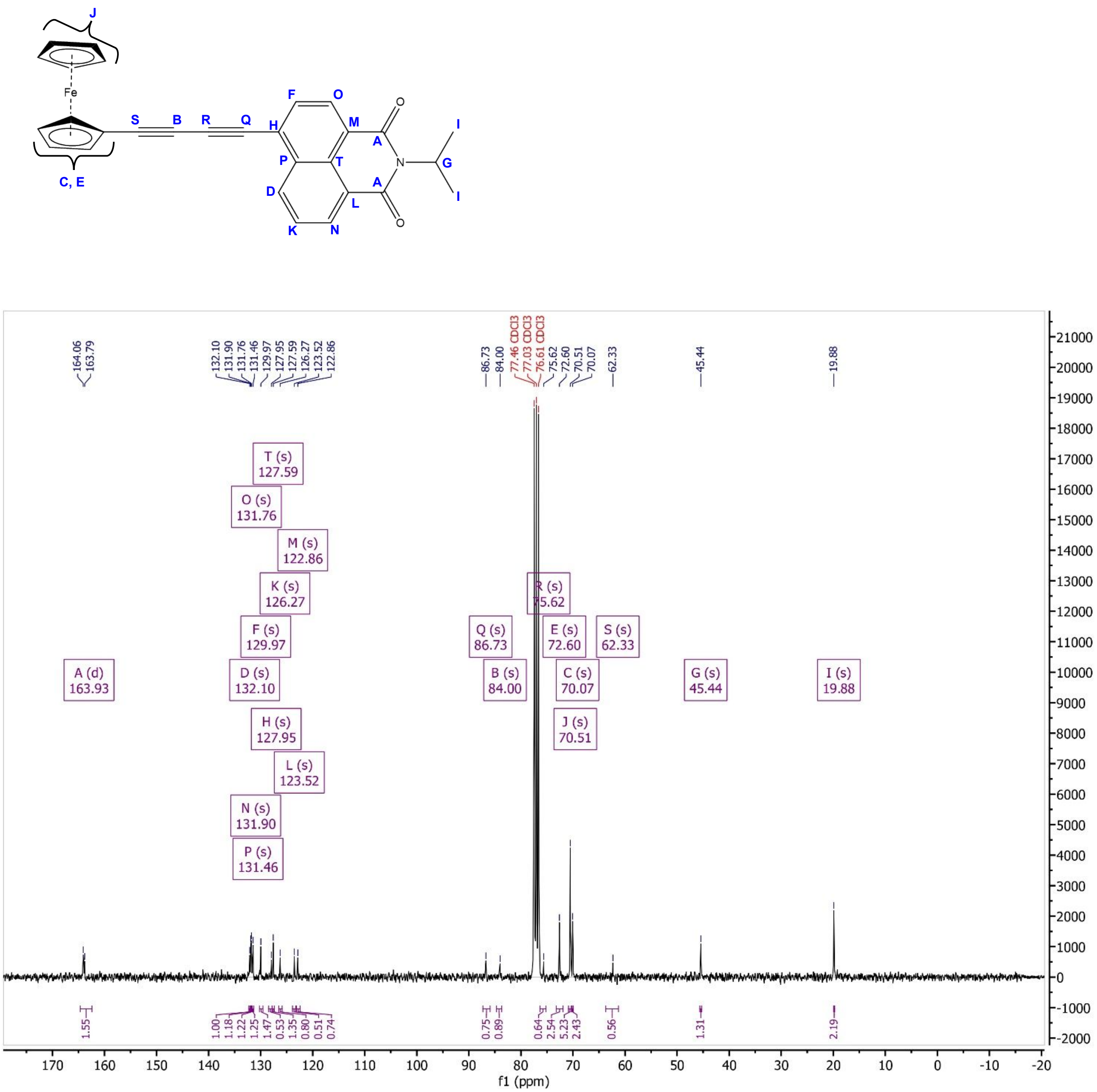

Figure S2. ^{13}C NMR Spectra of **Fc-C$_4$-NAP^{iPr}**. The peak labels are shown in inset.

S3. DFT computed ground state energies

DFT calculations show a very low energy barrier (ca. 0.39 k_BT) for rotation (Figure S3) which is lower than that for DC4NAP (ca. 0.97 k_BT). This enables the rotation of FcC4NAP which results in the participation of multiple conformers in ET dynamics of FcC4NAP.

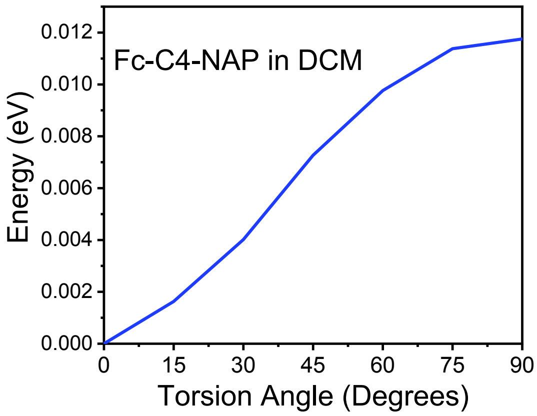

Figure S3. DFT calculated ground state energies for each conformer of FcC4NAP.

The natural transition orbitals analysis (Figures S4-S14) indicates that the acceptor-based locally excited state (LES) and the charge-separated state (CSS) are closely spaced in energy. The energy of the CSS is highly sensitive to the torsion angle between the NAP acceptor and the C4-tethered cyclopentadienyl ring of Fc, spanning a range of approximately 5000 cm^{-1}. Most of the low-energy states depicted in Figure 1 are associated with Fc, primarily involving the d orbitals of the Fe atom. Among the eight lowest-energy states, only two are linked to NAP: one corresponds to the LES, while the other represents the CSS.

S4. TD-DFT computed natural transition orbital analysis for selected angles

The natural transition orbitals (NTO) were determined for the transitions from ground states to the electronically excited states, as shown in Figures S4 to S14. The percentages shown on the arrows indicate the contribution to each transition.

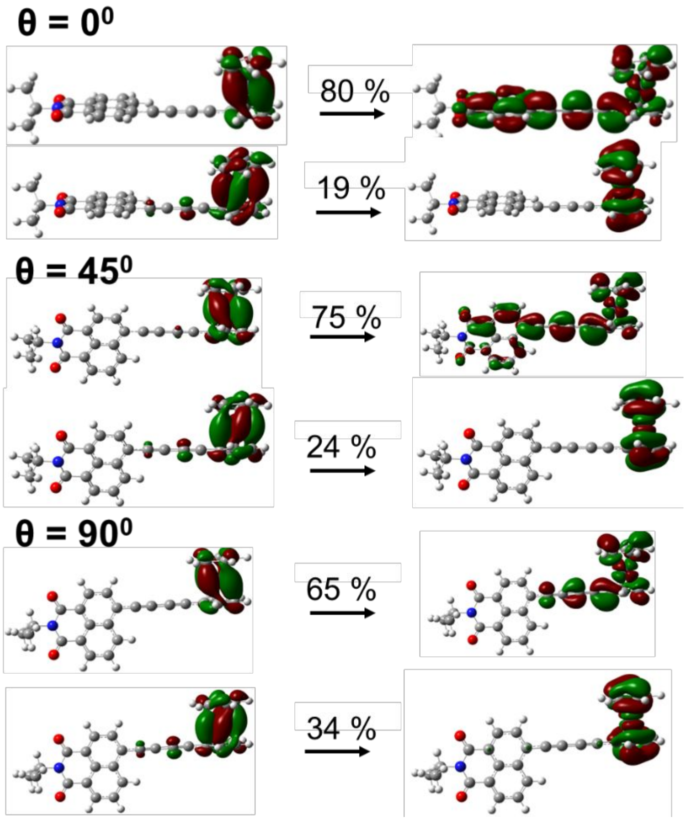

Figure S4. NTO analysis for the $S_0 \rightarrow S_1$ transition.

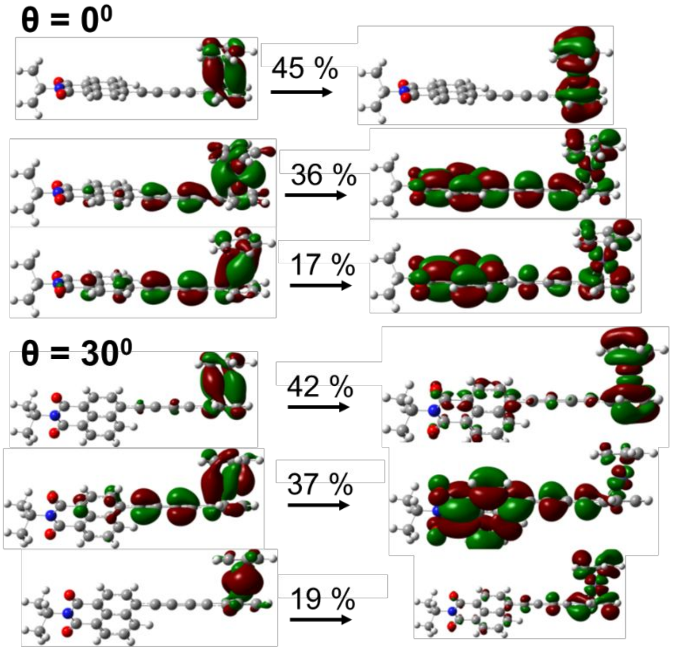

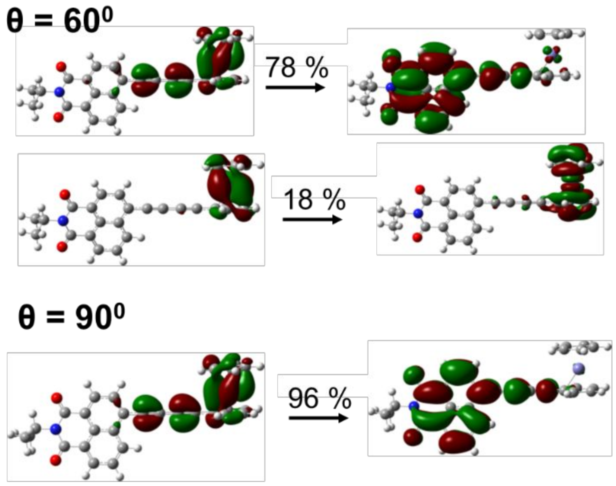

Figure S5. NTO analysis for the $S_0 \rightarrow S_3$ transition.

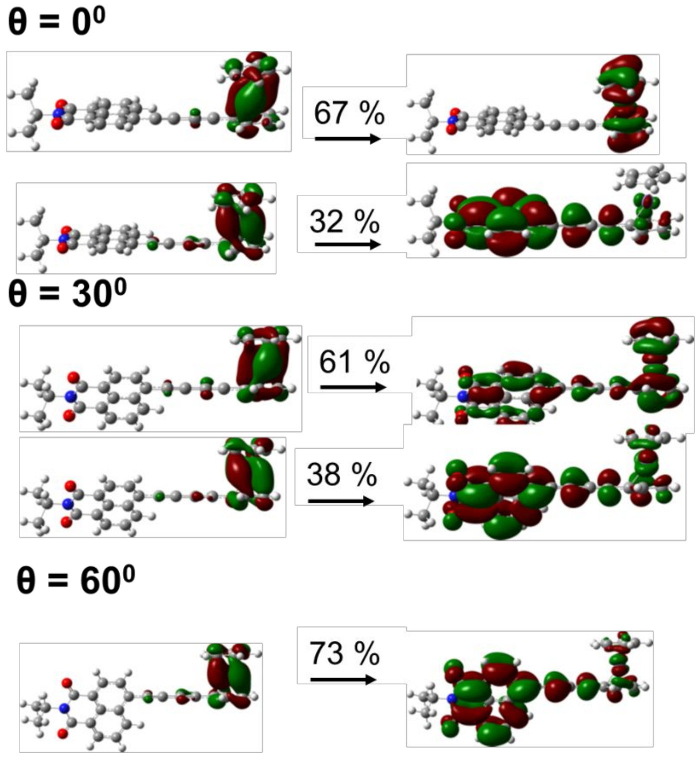

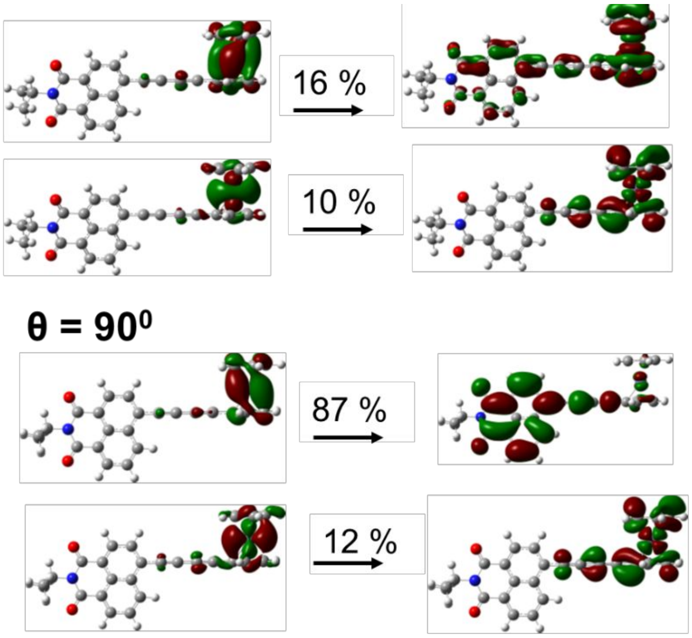

Figure S6. NTO analysis for the $S_0 \rightarrow S_4$ transition.

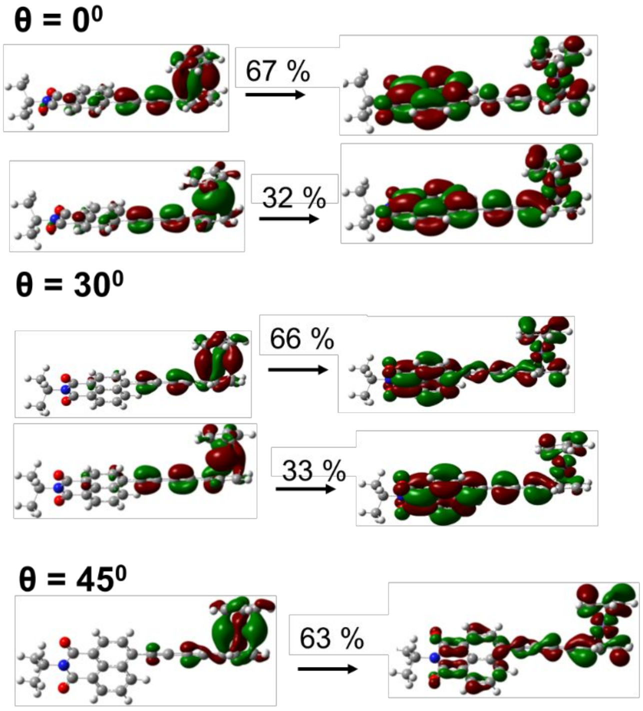

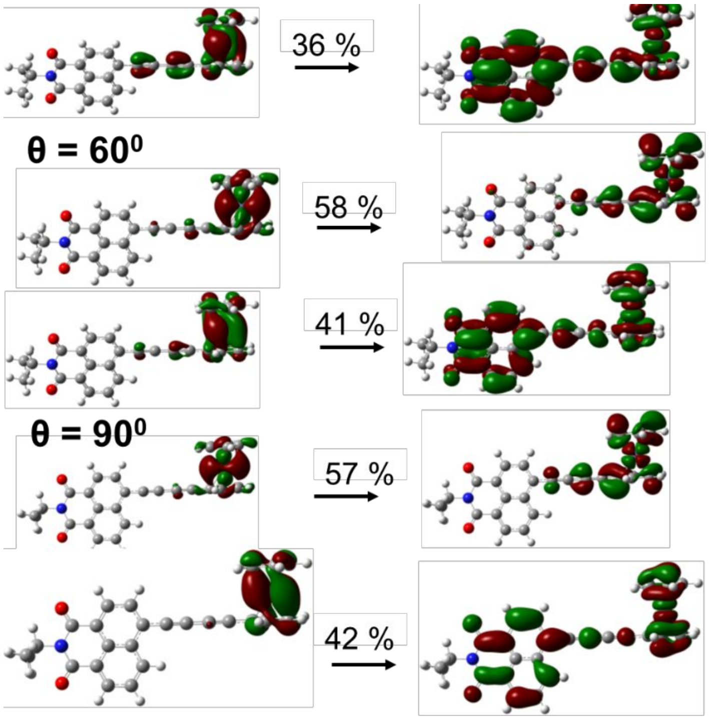

Figure S7. NTO analysis for the $S_0 \rightarrow S_5$ transition.

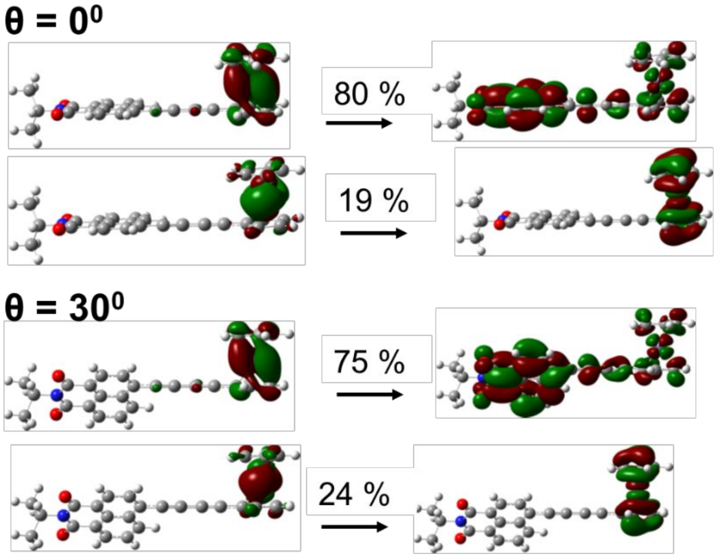

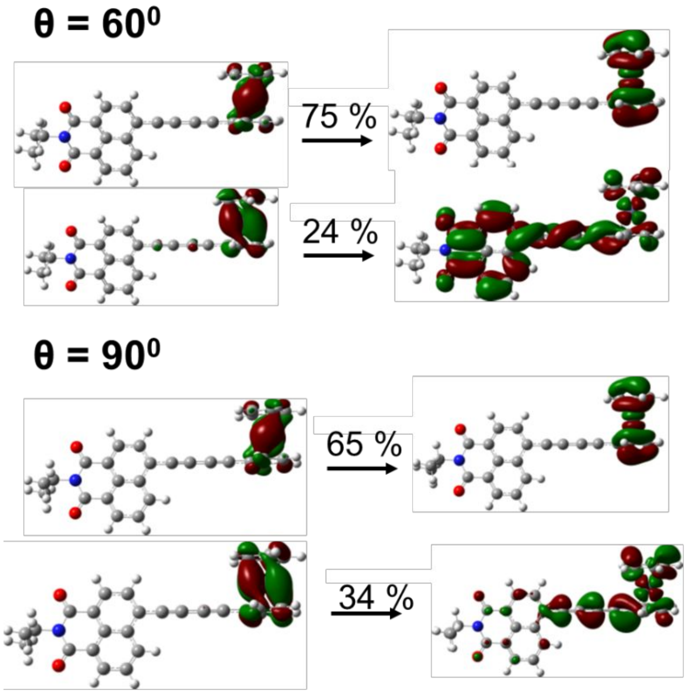

Figure S8. NTO analysis for the $S_0 \rightarrow S_6$ transition.

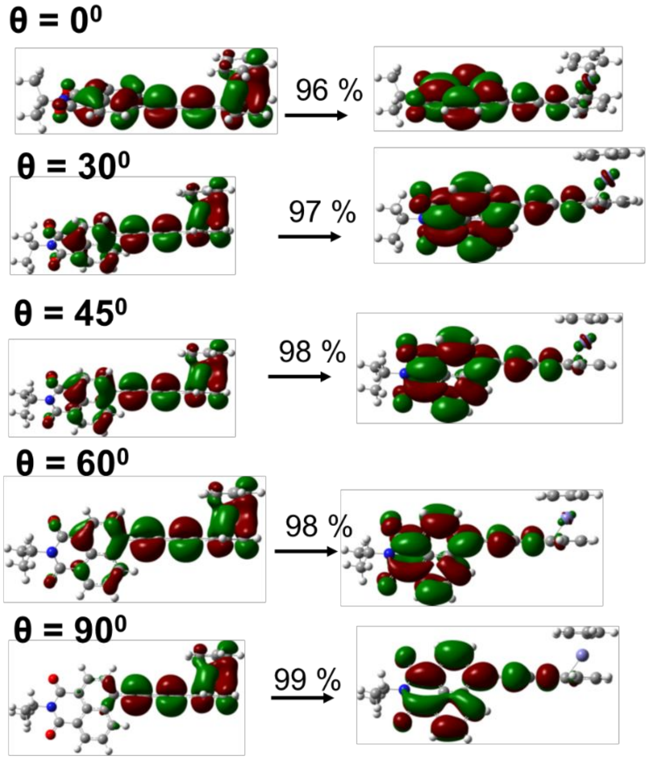

Figure S9. NTO analysis for the $S_0 \rightarrow S_7$ transition.

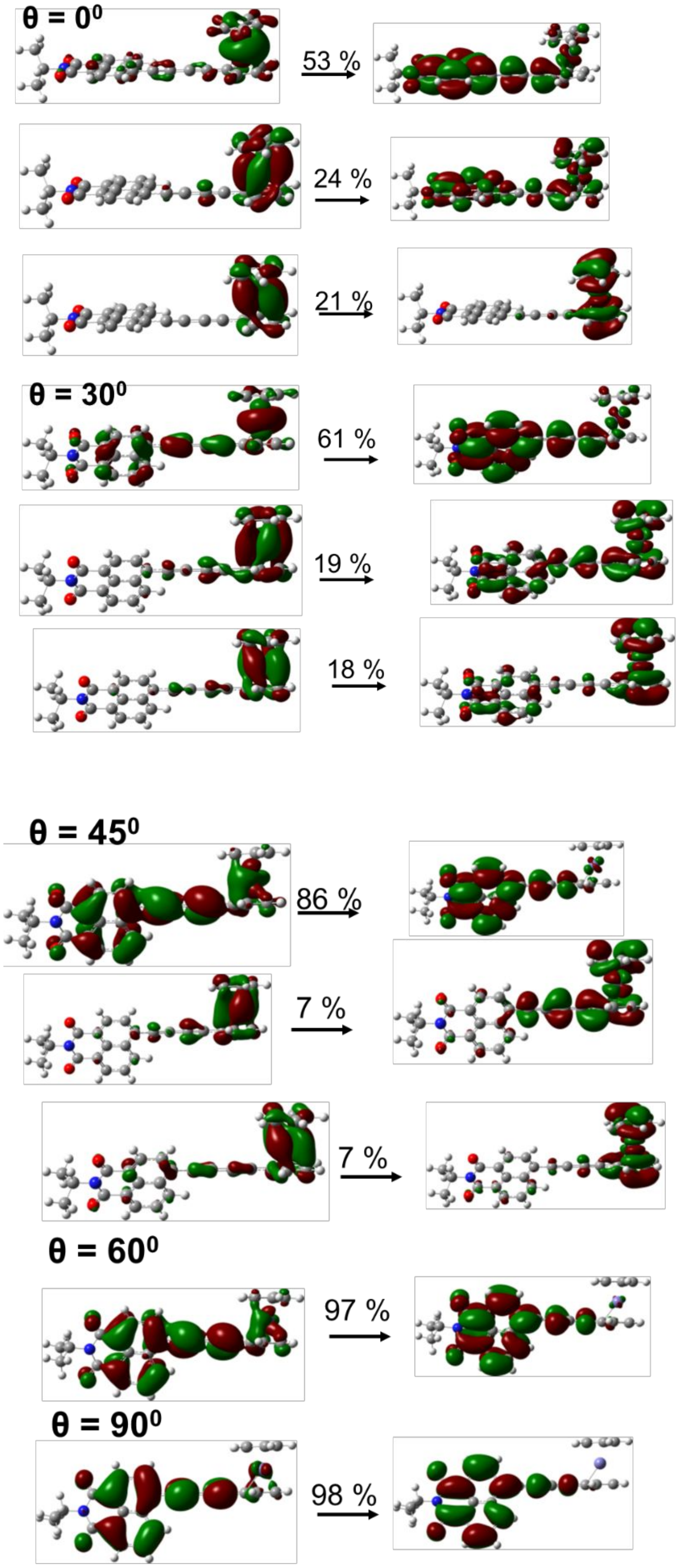

Figure S10. NTO analysis for the $S_0 \rightarrow S_8$ transition.

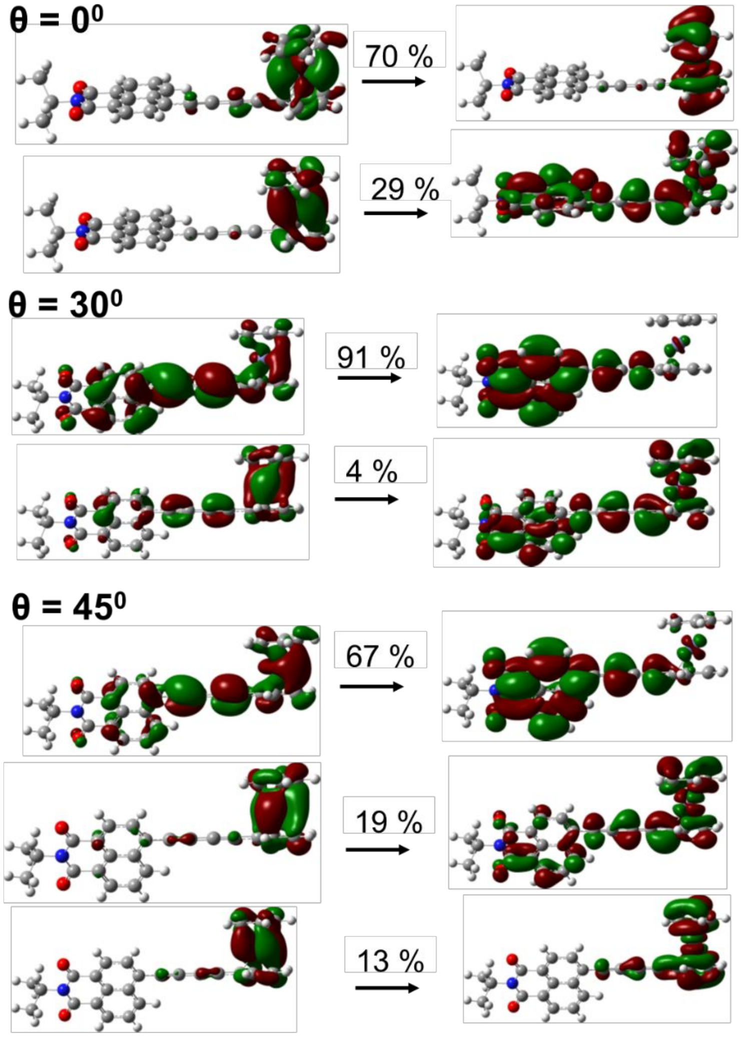

θ = 0⁰
70 %
29 %
θ = 30⁰
91 %
4 %
θ = 45⁰
67 %
19 %
13 %

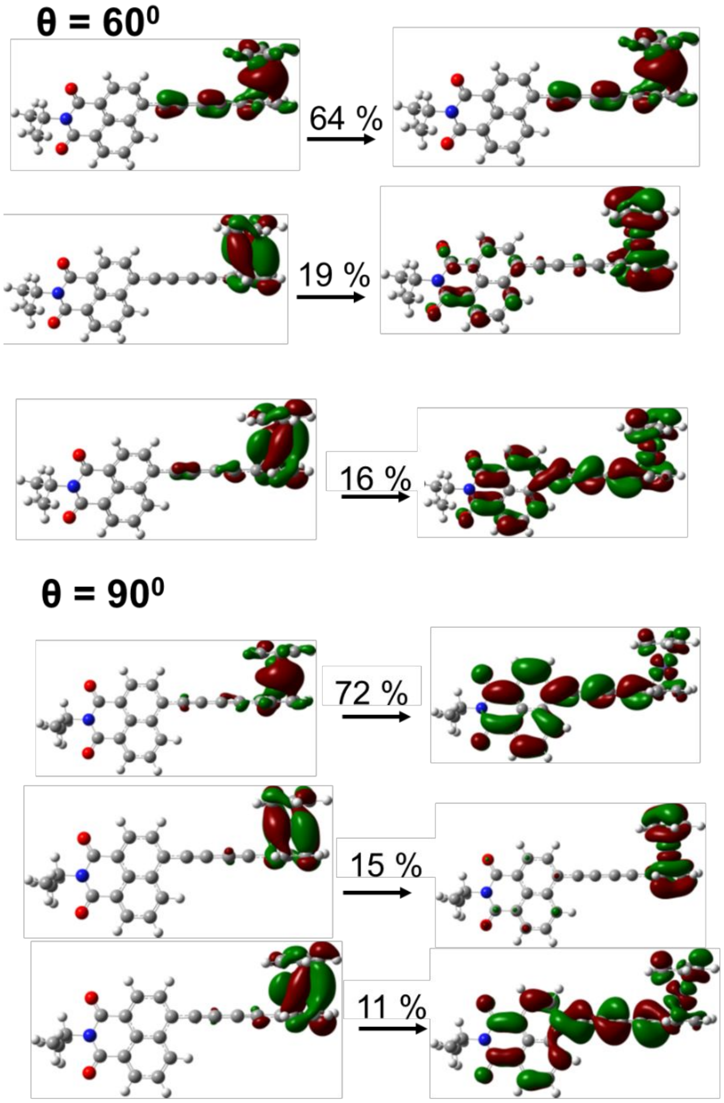

Figure S11. NTO analysis for the $S_0 \rightarrow S_9$ transition.

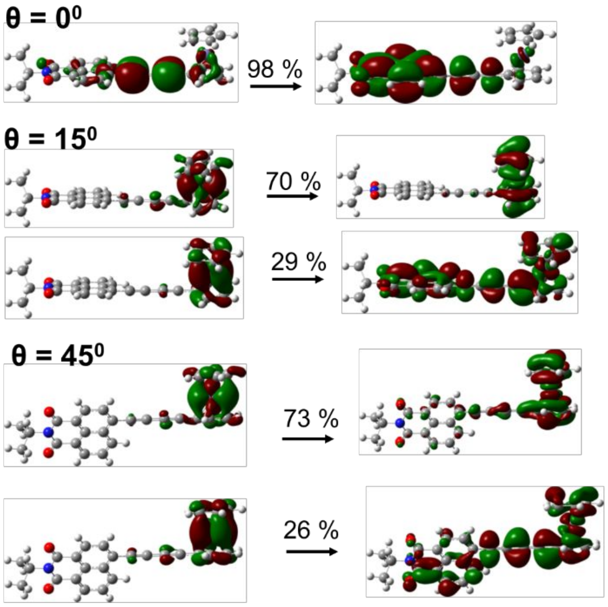

Figure S12. NTO analysis for the $S_0 \rightarrow S_{10}$ transition.

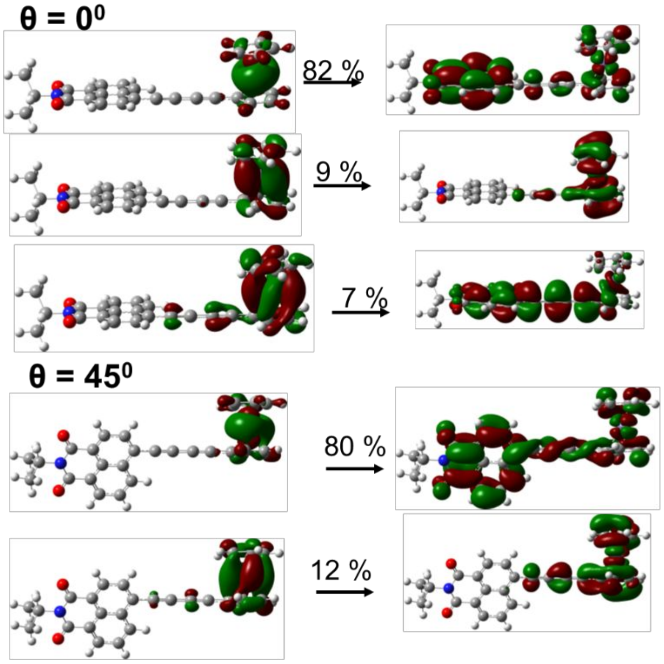

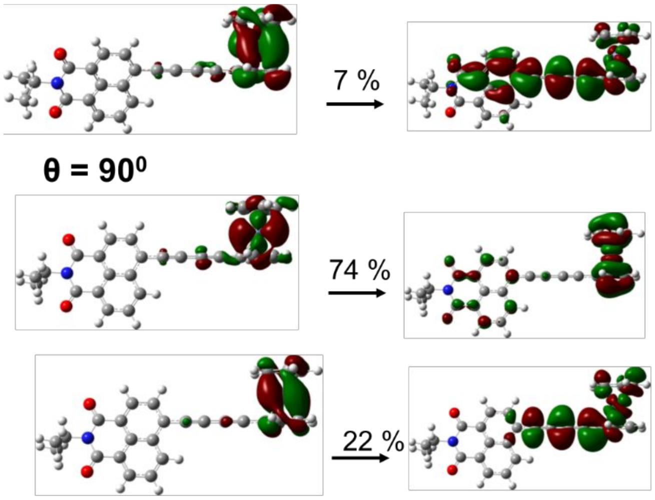

Figure S13. NTO analysis for the $S_0 \rightarrow S_{11}$ transition.

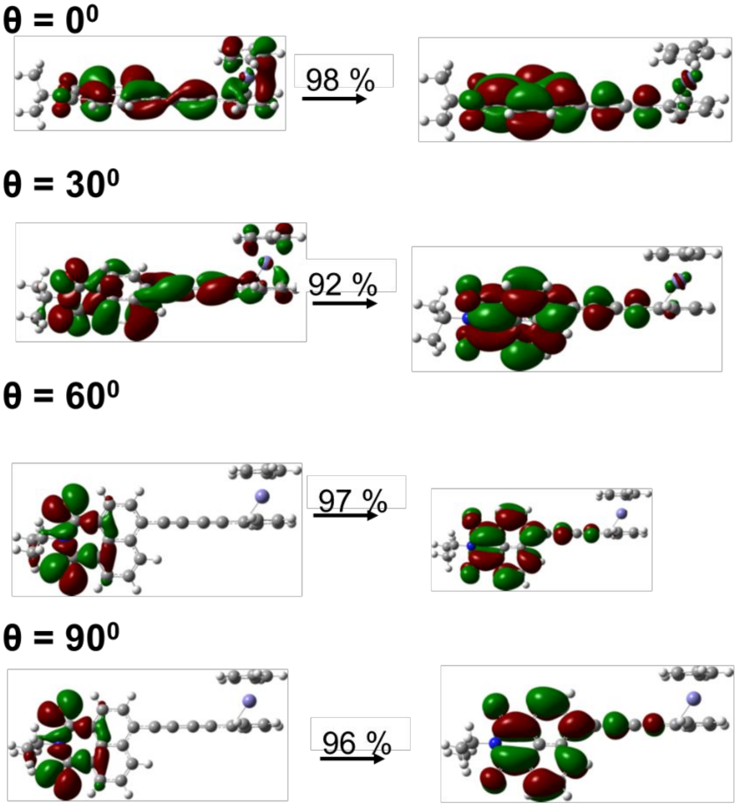

Figure S14. NTO analysis for the $S_0 \rightarrow S_{12}$ transition.

S5. TD-DFT modeled linear absorption spectrum

Linear absorption spectrum of Fc-C4-NAP was modeled using the TD-DFT computed energies and oscillator strengths of the electronic states from S1 to S16 as shown in Figure S15. The influence of various

torsional conformations was evaluated using Boltzmann weighting. To achieve closer agreement with experimental results, the computed line spectra were broadened using Gaussian functions with slightly different widths for each set (dotted black line).

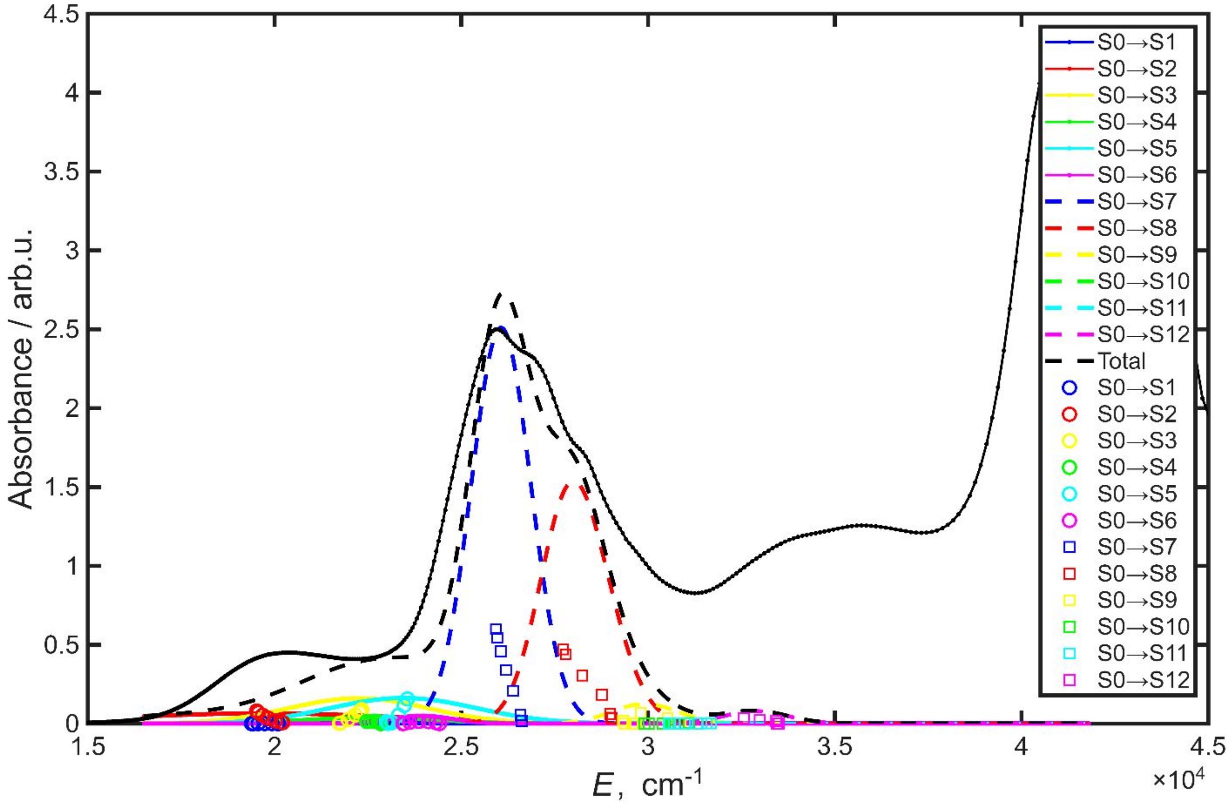

Figure S15. Absorption spectrum of Fc-C4-NAP in DCM (black line) and its modeling using DFT computed frequencies and oscillator strengths for the states S1 to S12 shown as symbols. Sets of circles of the same color show the frequencies and oscillator strength (Y-axis, arbitrary but comparable between the sets) computed for different torsion angles of (0, 15, 30, 45, 60, 75, and 90 degrees). The contributions of different torsion conformations were computed using the Boltzmann factor. The line spectra were broadened with Gaussian functions of slightly different widths for different sets (dotted black line) to better match the experiment. The computed contributions from each state are shown as dotted lines.

S6. Variation of oscillator strength with torsion angle

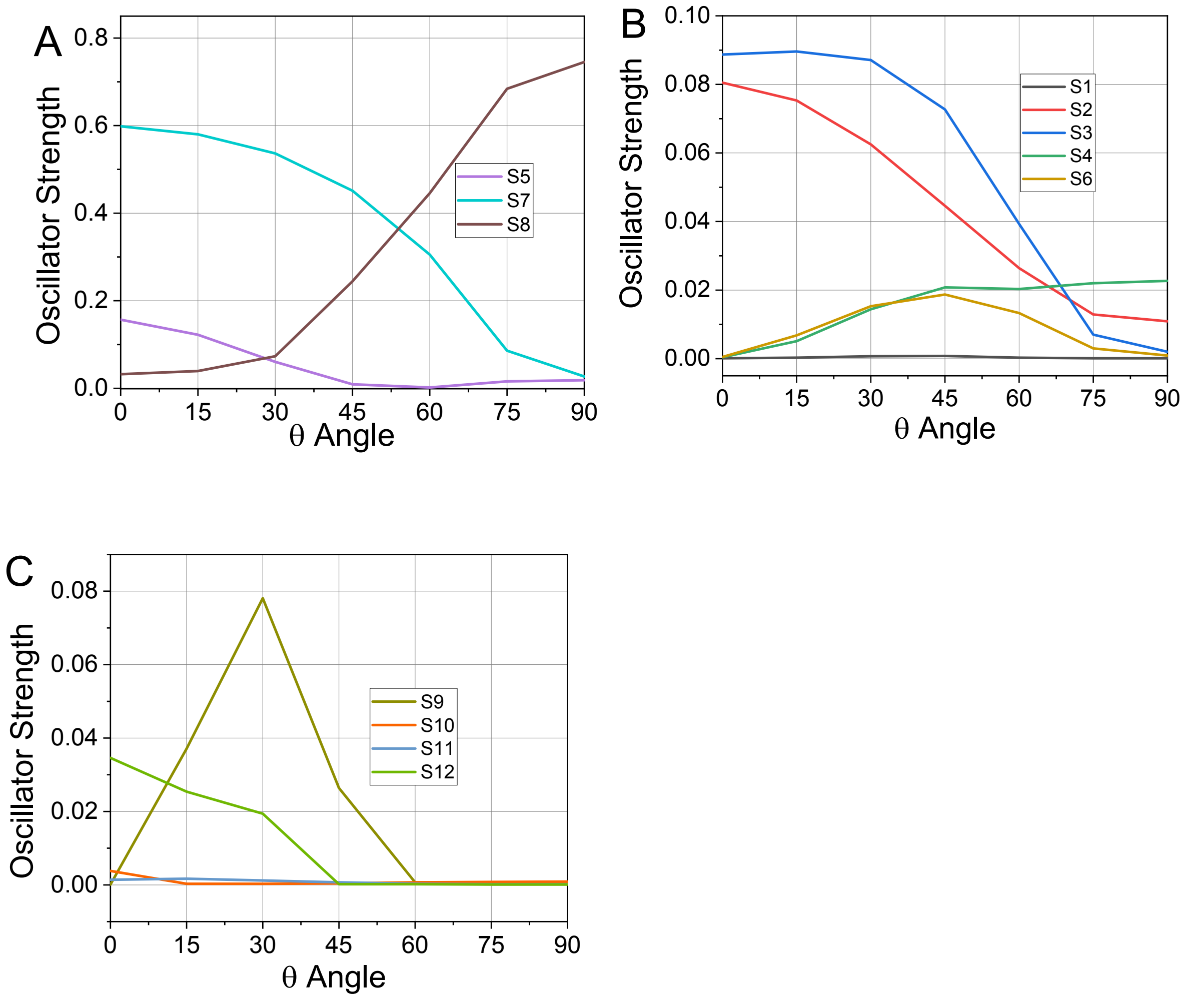

Figure S16. Variation of oscillator strength with torsion angle for different electronic states: (A) higher oscillator strength states (S5, S7, S8); (B) lower oscillator strength states (S1–S4, S6); and (C) higher excited states (S9–S12).

S7. Photoinduced dynamics of Fc-C4NAP in toluene.

The similarity of the spectral features in the corresponding UV/Vis, UV/mid-IR, and UV/Vis DAS spectra of Fc–C4–NAP in DCM and toluene indicates that the photoinduced dynamics are largely comparable in both solvents. The near similarity between the corresponding DAS also justifies the similarity of the relaxation processes involved. The slightly longer characteristic times observed in toluene are likely due to its lower polarity relative to DCM. UV/mid-IR spectra also possess similar behavior with the delay. Focusing on the triple bond region, a small and gradual blue shift towards higher frequency can be observed at delay times exceeding 5 ps, as for Fc-C4-NAP in DCM (Figure 3D). Moreover, both the positive and negative spectral features decay to zero at 100 ps.

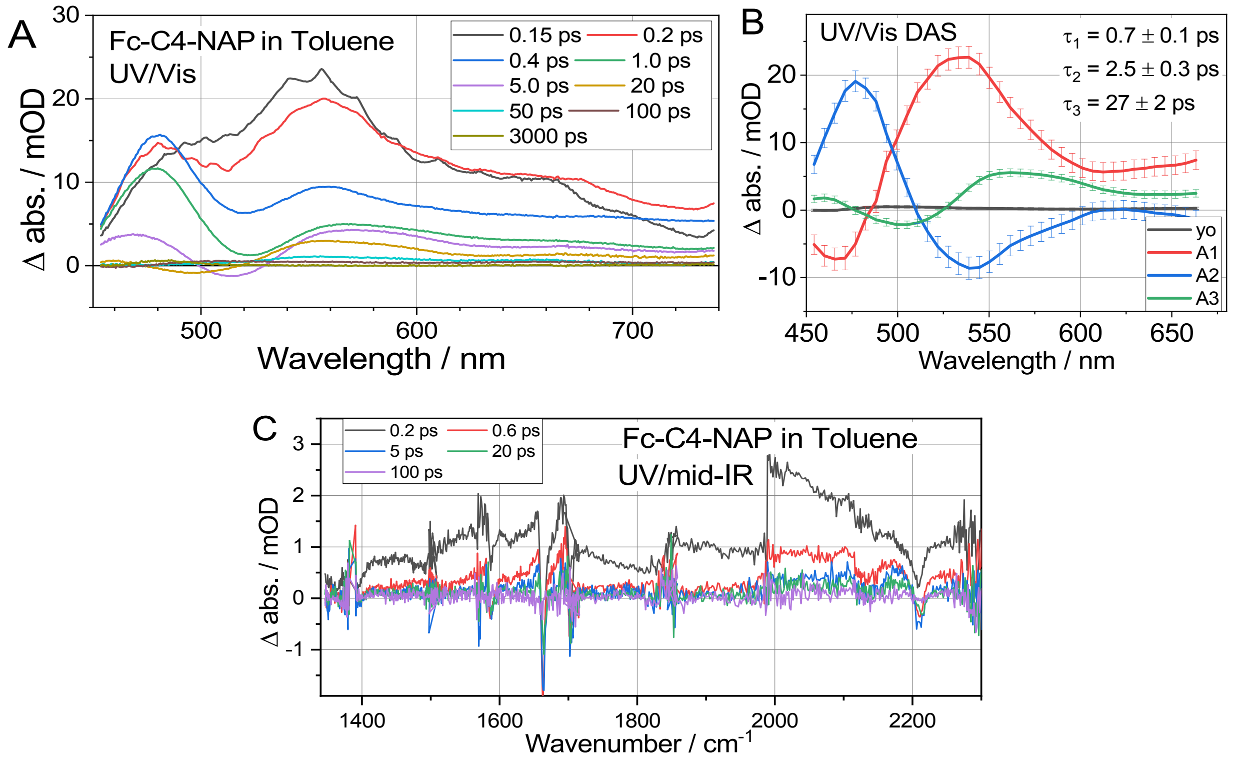

Figure S17. Transient UV/Vis (A) and UV/mid-IR (C) spectra for Fc-C4-NAP in toluene at indicated delay times. Pannel B shows UV/Vis DAS with time components indicated as inset.
